# Nanodrugs Targeting T Cells in Tumor Therapy

**DOI:** 10.3389/fimmu.2022.912594

**Published:** 2022-05-25

**Authors:** Maximilian Haist, Volker Mailänder, Matthias Bros

**Affiliations:** University Medical Center Mainz, Department of Dermatology, Mainz, Germany

**Keywords:** nanodrug, nanoparticle, T cell, regulatory T cell, immunotherapy, CAR-T cell, mRNA, melanoma

## Abstract

In contrast to conventional anti-tumor agents, nano-carriers allow co-delivery of distinct drugs in a cell type-specific manner. So far, many nanodrug-based immunotherapeutic approaches aim to target and kill tumor cells directly or to address antigen presenting cells (APC) like dendritic cells (DC) in order to elicit tumor antigen-specific T cell responses. Regulatory T cells (Treg) constitute a major obstacle in tumor therapy by inducing a pro-tolerogenic state in APC and inhibiting T cell activation and T effector cell activity. This review aims to summarize nanodrug-based strategies that aim to address and reprogram Treg to overcome their immunomodulatory activity and to revert the exhaustive state of T effector cells. Further, we will also discuss nano-carrier-based approaches to introduce tumor antigen-specific chimeric antigen receptors (CAR) into T cells for CAR-T cell therapy which constitutes a complementary approach to DC-focused vaccination.

## Introduction

So far, the vast majority of nano therapeutics applied for tumor therapy aims to target either tumor cells for direct killing by delivery of cytotoxic drugs (1) or antigen presenting cells (APC) like dendritic cells (DC) to evoke tumor antigen-specific T cell responses by co-delivery of antigen and adjuvant (2). The latter, immunotherapeutic nano vaccination approach is quite attractive since DC, which are considered the most potent APC due to their capacity to induce even primary T cell responses, are specialized in the uptake of exogenous material *via* several receptors allowing endocytosis and phagocytosis (3). Further, APC are equipped with numerous cell surface as well as intracellular receptors that sense danger signals (4). Nano-vaccines can be considered as minimal pathogens, providing a source of antigen and adjuvant, i.e. APC-stimulating danger signals.

However, this vaccination approach is limited by immunoregulatory immune cells, including myeloid-derived suppressor cells (MDSC) as well as regulatory T cells (Treg), that are induced and expanded by molecular cues of the tumor as well as of accessory cells like tumor-associated macrophages (TAM) and cancer-associated fibroblasts (CAF), forming the tumor microenvironment (TME) (5). MDSC and Treg are able to spread tumor tolerance by imprinting a pro-tolerogenic state in APC, by inhibiting activation of (naïve) T cells, and by deactivating T effector cells (6, 7). Therefore, the development of nanodrugs that address and either deplete or reprogram immunoregulatory cell types have come into focus (8). Whereas MDSC and TAM are equipped with numerous uptake receptors and thereby may readily internalize nanodrugs, especially T cells have proven more difficult in that regard. Therefore, approaches employing nanodrugs to induce T cell activation have so far only scarcly been applied.

**Figure 1:**
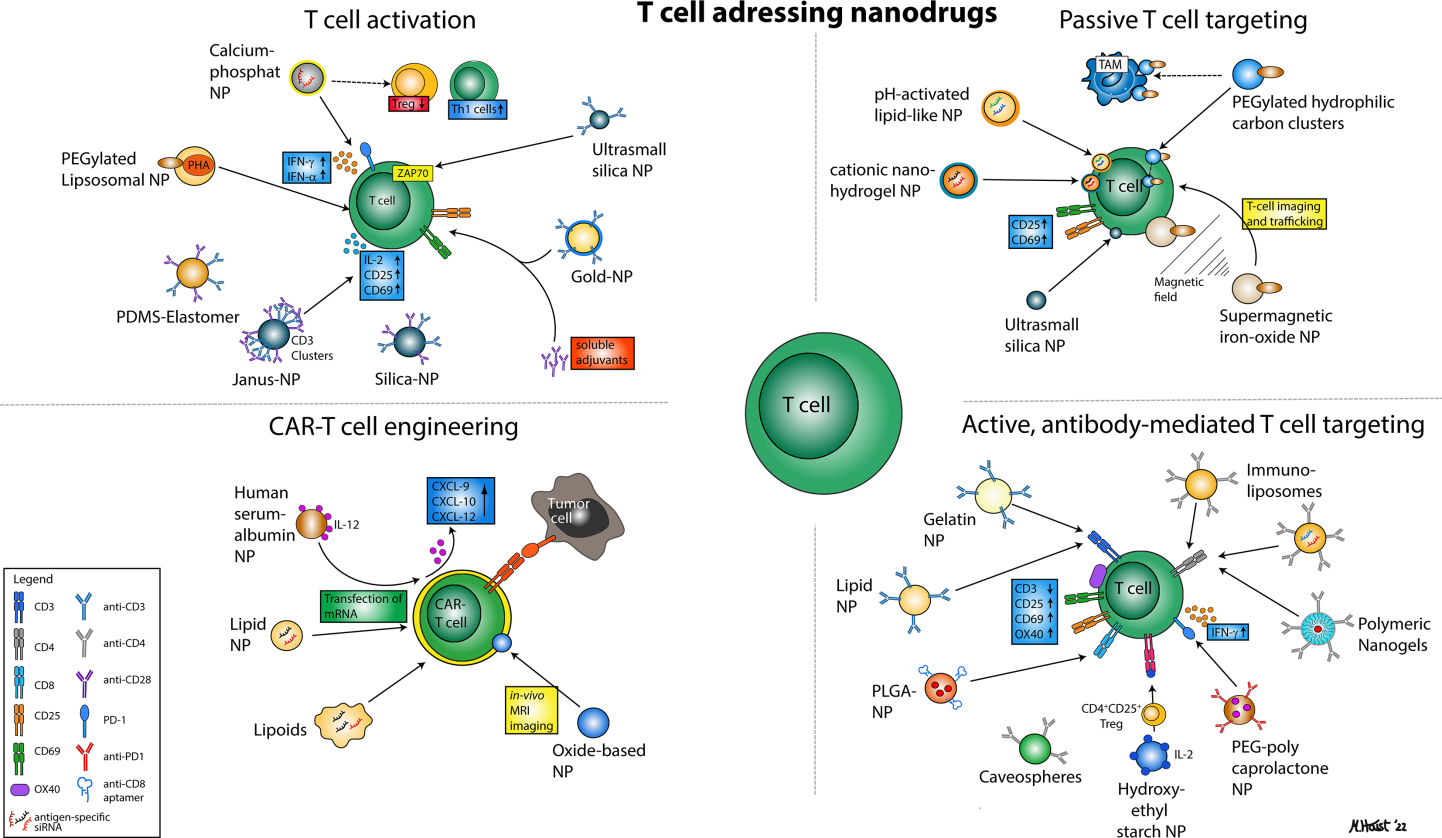
Illustration of various nanoparticular approaches to address T cells for immunotherapy. In general, four major mechanisms can be distinguished that comprise nanodrugs aiming to activate T cells (1), nanodrugs that allow for passive (2) or active, antibody-mediated T cell targeting (3) and nanodrugs that may enhance the efficacy of CAR T cell therapies.

This review aims to present current nanodrug-based strategies developed to address and modulate T cells (an overview is given in **Figure 1**). In most cases, according studies have focused on the activation and expansion of T effectors cells and the generation of chimeric antigen receptor (CAR-)T cells, genetically altered to express a defined tumor antigen-specific T cell receptor. Both approaches focus on expanding the pool of tumor antigen-specific T cells. In contrast, the complementary strategy to reprogram Treg towards T effector cells by nanodrugs has been issued in a limited number of studies only.

So far, most clinical studies focused on the generation and adoptive transfer of CAR-T cells, while mRNA-based nanodrugs directly targeting T cells have not been translated widely into the clinic. However, a number of clinical trials have applied immune-checkpoint inhibitors (ICI) in combination with nanodrugs in order to indirectly address the T cell compartment and induce a robust anti-tumor T cell response. Most commonly, these indirect approaches act *via* the induction of immunogenic cell-death (ICD), the depletion of immunosuppressive cell types and DC-mediated T cell activation.

## T Cell Activation

For *in vitro* activation of T cells in the context of adoptive T cell transfer, several types of nano structures have been developed to improve polyclonal stimulation with immobilized agonistic anti-CD3 and CD28-specific antibody. In many cases the T cell stimulatory antibodies are attached to nano-sized beads, termed artificial (a)APC (9).

Matic and coworkers observed an inverse relation between the density of immobilized anti-CD3 antibody and the extent of primary human CD4^+^ T cell activation as reflected by CD69 expression and IL-2 levels (10). T cell proliferation required the co-application of anti-CD28 antibody, which was applied in soluble form. Later, beads that were conjugated with agonistic anti-CD3 and -CD28 antibodies were introduced for T cell stimulation, which displayed elevated stimulation properties due to the structural similarity of the stimulatory beads and APC (11). This concept was developed further by using polydimethylsiloxane (PDMS) elastomer as a carrier substrate, which was coated with albumin for biocompatibility (12). According beads (Ø 28-40 nm) induced faster and longer-lasting T cell divisions as compared to antibody-coated `stiff´ beads of comparable size, namely DynaBeads and polystyrene NP. This demonstrates that besides the density of the stimulating agents also physical properties of nano-carriers like stiffness/softness of the underlying material are important to optimize T cell stimulation.

The activation of primary CD4^+^ T cells was also improved by employing oligo peptides that enhanced cell adhesion (13). To this end, PEG hydrogel was conjugated with fibronectin-derived peptides, known to be engaged by integrin receptors expressed by T cells (14). These functionalized PEG hydrogels were decorated in addition with Au-NP, which in turn were conjugated with agonistic CD3-specific antibody. Agonistic anti-CD28 antibody was applied in soluble form. CD4^+^ T cell activation (CD69 expression, IL-2 production) and proliferation was improved in the presence of the integrin-binding Arg-Gly-Asp tripeptide (15), and correlated inversely with Au-NP density on PEG hydrogels (minimal distance: 20 nm). In a follow-up study T cell activation and proliferation was further increased by applying a cocktail of phorbol 12-myristate 13-acetate (PMA), ionomycin and Golgi inhibitors (16) that enhanced IL-2 production *via* activation of protein kinase C and elevation of cytosolic calcium levels (17).

Recently, Hammink and coworkers showed that as compared to direct conjugation of agonistic CD3 and CD28 antibodies to the bead surface the inclusion of semi-flexible polyisocyanopeptide polymer linkers of about 400 nm in length enhanced the activation of primary human CD4^+^ and CD8^+^ T cells in terms of proliferation in terms of proliferation, as well as the production of IL-2 and interferon (IFN)-γ production (18).

Whereas in the aforementioned approach as well as in case of standard aAPC both types of agonistic antibody are evenly distributed, Lee and Yu asked for the potency of aAPC based on silica NP (Ø ~500 nm) on which anti-CD3 antibody was clustered in segregated patches, surrounded by anti-CD28 antibody (19). These so-called Janus NP conferred much stronger T cell activation as in case of homogenous antibody distribution.

NP may exert intrinsic T cell stimulatory activity as shown for ultrasmall silica NP (Ø <10 nm) that engaged the T cell receptor and CD3, and thereby induced ζ-chain-associated protein kinase 70 (ZAP70) phosphorylation as well as induction of activation markers (CD25, CD69) (20). However, T cell proliferation and IL-2 production required the co-application of agonistic CD28 antibody or PMA.

The lectin phytohemagglutinin (PHA) stimulates T cells by cross-linking of the T cell receptor (21), but is highly toxic (22). Most recently, Alhallak and coworkers showed that PEGylated liposomes, comprised of DPPC and cholesterol, allowed efficient loading with PHA (23). These liposomes conferred strong T cell activation *in vitro*. In contrast to soluble PHA, the liposomal PHA formulation exerted no increased toxicity *in vivo*, and mediated T cell activation as well. Further, in a murine multiple myeloma tumor model, *in vivo* application of liposomal PHA resulted in a strong decrease in tumor growth, which has been attributed to T cell activation. It was not clarified, however, which additional cell types engaged liposomal PHA and were affected by the cargo.

## T Cell Targeting

Some types of nanoparticles (NP) have been demonstrated to possess an intrinsic T cell targeting property, termed passive targeting. However, in a growing number of studies active T cell targeting has been achieved by conjugation of surface receptor binding ligand derivatives or antibodies.

## Passive

In a proof of concept study we issued the potential of non-functionalized NP for uptake by T cells (24). To this end, we compared binding and uptake of polystyrene particles of different sizes (Ø 63-121 nm) and surface functionalization by human pre-activated CD4^+^ and CD8^+^ T cells. In the course of these experiments amino-functionalized polystyrene beads (Ø 65 nm) were most efficient as evaluated by confocal microscopy. NP were apparent in membrane-surrounded vesicles. Of note, we observed a considerable release of NP after longer periods of incubation. NP uptake exerted no detrimental effects on cell viability. Interestingly, NP/T cell interaction was attenuated in the presence of human serum in a dose-dependent manner. Both IFN-γ production (CD4^+^ and CD8^+^ T cells) and target cell killing (CD8^+^ T cells) were not affected at low to intermediate NP concentrations.

The potential of superparamagnetic iron oxide NP (SPION) to label T cells *in vitro* in the context of adaptive T cell transfer for tumor therapy was assessed in several studies. For example, Liu and coworkers demonstrated that PEG-coated SPION could be used to efficiently address T cells without causing any detrimental effects on their physiological functions (25). SPION-labeled T cells could be tracked *in vivo* by magnetic resonance imaging (MRI) as shown in a rodent transplantation model. Besides *in vivo* tracking, SPION-labeled pre-isolated CD3^+^ T cells that were labeled *ex vivo* with SPION and systemically injected could be directed *in vivo* e.g. to tumor sites by applying a magnetic field as shown in several proof of concept studies (26, 27). Polyclonal T cell activation increased SPION (Ø 54 nm) binding, but also resulted in a drop in overall T cell viability.

Huq and coworkers demonstrated specific uptake of poly(ethylene glycol) (PEG) functionalized hydrophilic carbon clusters (HCC) by splenic rat T cells *in vivo* after systemic application (28), but not by lymph node T cells. Subsequent *in vitro* spleen cell studies corroborated these findings and showed that both CD4^+^ and CD8^+^ T cells internalized PEG-HCC by clathrin-mediated endocytosis both at resting state and at enhanced level after mitogenic stimulation. In accordance with the previously reported scavenger activity of PEG-HCC (29), accordingly treated T cells contained lower levels of oxygen radicals, and displayed lower activation in response to antigen-specific stimulation (28). Interestingly, myeloid cell types displayed considerable binding, but no uptake of PEG-HCC. Accordingly, macrophages were not affected in terms of activation. In contrast to PEG-HCC, ultra-small silica NP Staff(Ø 3.6-5.1 nm) displayed no T cell-specific uptake when assayed with human peripheral blood mononuclear cell (PBMC) preparations, but conferred strong T cell activation (30). In this regard, CD4^+^ and CD8^+^ T cells displayed strong upregulation of surface activation markers (CD25, CD69) and IFN-γ release, whereas Th2-associated cytokines were not induced. Of note, T cell activation did not result in T cell proliferation. Somewhat surprisingly, we observed an inverse correlation between the size of silica nanocapsules (SiNC; Ø 50-400 nm) and their cytotoxic effect on human primary CD8^+^ T cells (31). Therefore, we chose SiNC of larger size for subsequent experiments. We demonstrated that SiNC (Ø 248 nm) were internalized by human primary CD8^+^ T cells, and successfully transferred siRNA specific for the T cell inhibitory receptor programmed cell death protein (PD-)1 (32). The downregulation of PD-1 was accompanied by upregulated expression of the surface activation markers CD25, CD69 and CD71. Besides SiNC we also assayed other types of NP for their ability to transfect T cells with siRNA, namely lipophilic triphenylphosphonium cation-modified diblock copolymer structures with a terminal 3-guanidinopropyl methacrylamide block for the complexation of siRNA, and cationic nanohydrogel NP (Ø <28 nm) (33). The latter were based on an amphiphilic block copolymer harboring a hydrophilic block, which consisted of triethylenglycol methylether methacrylate and a hydrophobic block of pentafluorophenyl methacrylate as reactive ester. The derived amphiphilic polymers self-assembled into polymeric micelles with a reactive ester core, substituted from hydrophobic to cationic by using bi-functional amine containing cross-linkers. Primary human CD8^+^ T cells isolated from PBMC and stimulated with agonistic anti-CD3 antibody and IL-2 showed uptake of either siRNA carrier.

Whereas the aforementioned carrier systems have been evaluated to alter T cell activation by RNA interference using siRNA, Tanaka and coworkers optimized lipid NP for mRNA transfection of T cells using the human CD4^+^ T cell line Jurkat as a model (34). Stepwise improvements resulted in a formulation consisting of SS-cleavable and pH-activated lipid-like material (ssPalmO-Phe-P4C2), previously shown to promote endosomal escape and release of mRNA into the cytoplasm (35), 1-palmitoyl-2-oleoyl-sn-glycero-3-phosphatidylethanolamine, cholesterol and 1-(monomethoxy PEG2000)2,3-dimyristoylglycerol.

Taken together, the aforementioned studies confirmed that nanodrugs may successfully deliver cargo into (activated) T cells. However, the obstacle of competitive uptake of NP by other types of immune cells with more pronounced endocytic activity, thereby interfering with T cell -specific targeting, was scarcely addressed.

## Active

In order to specifically address T cells and to enhance NP uptake, a number of approaches evaluated T cell-specific targeting by addressing suitable surface receptors like CD3 and CD4 with according antibodies.

Dinauer and coworkers demonstrated that targeting of the pan-T cell marker CD3 using gelatin NP coated with anti-CD3 antibody yielded strong uptake of that functionalized NP by T cell leukemia cells as well as primary T cells *in vitro* (36). Most recently, T cell-specific transfection with mRNA *in vivo* was achieved by using lipid NP containing an ionizable cationic lipid (DLin-MC3-DMA), that was conjugated with a CD3-specific antibody (37). Intravenous application resulted in an accumulation of fluorescent reporter mRNA containing lipid NP in the spleen, and a transfection efficiency of up to 4% of all splenic and 7% of circulating T cells. In accordance with the agonistic property of the CD3 antibody, transfected T cells expressed activation markers (CD25, CD69, OX40), accompanied by downregulation of CD3ε, and displayed elevated plasma levels of T cell-associated cytokines. Further, when injected into tumor-bearing mice undergoing immunotherapy, transfected T cells were apparent in the tumor and tumor-draining lymph nodes.

Several studies aimed to address CD4^+^ T cells by NP conjugated with CD4-binding antibodies. Ramana and coworkers evaluated the efficiency of this targeting strategy by using anti-CD4 antibody conjugated immunoliposomes consisting of phosphatidyl choline (PC), distearoylphosphatidylethanolamine (DSPE)–PEG), 1,2-dipalmitoyl-sn-glycero-3-phosphoethanolamine-N-(7-nitro-2-1,3-benzoxadiazol-4-yl) and cholesterol (38). These immunoliposomes were loaded with two anti-retroviral drugs (Ø ~71 nm). CD4-targeting immunoliposomes were efficiently internalized by cells of the Jurkat T cell line, and both drugs exerted biological activity. CD4-targeting lipid NP were also employed to deliver CD45-specific siRNA into murine primary CD4^+^ T cells (39). *In vivo* administered lipid NP, consisting of cholesterol, distearoylphosphatidylcholine, 1,2-distearoyl-sn-glycero-3-phosphoethanolamine-PEG, and Dlin-MC3-DMA (40), with a diameter of ~130 nm, were detectable in various organs only in CD4^+^ T cells, and caused CD45 downregulation, which was associated with a decrease in CD4 surface levels due to endocytosis-mediated sequestration (41). Recently, Tombácz and coworkers demonstrated efficient mRNA transfection of murine CD4^+^ cells *in vivo* after intravenous injection using CD4 antibody-conjugated lipid NP consisting of the proprietary ionizable cationic lipid ALC-0307 (42), PC, cholesterol and PEG-lipid NP (43). To demonstrate efficacy of their lipid NP in vivo, reporter mice were used which were engineered to express a fluorescent protein after Cre recombinase mediated recombination. When injecting these reporter mice with complexes composed of Cre recombinase encoding mRNA and lipid NP high frequencies of CD4+ T cells (spleen: ~ 60 %, lymph nodes: ~ 40 %) were found to express the fluoresecent reporter. Canakci and coworkers used anti-CD4 antibody conjugated polymeric nanogels generated by polymerization of monomeric PEG methyl ether methacrylate (PEGMA) and pyridyl disulfide ethyl methacrylate to deliver the tubulin inhibitor mertansine to T cell lymphoman cell lines (44).

Aptamers are peptides or oligonucleotides which are identified by library screenings to bind a given target molecule at high affinity and are much smaller than according antibodies (45). The aptamer CD8AP17 specifically binds human CD8 (46). Mansouri and coworkers used poly(lactic-co-glycolic acid) (PLGA) based NP that were modified with chitosan to promote endosomal escape (47), conjugated with the aptamer CD8AP17 (Ø 345 nm) to deliver the calcineurin inhibitor tacrolimus (48) specifically to cells of an immortalized CD8^+^ T cell line (49). Glass and coworkers generated so-called caveospheres, which consist of self-assembling Caveolin-1 fused with Staphylococcal IgG Fc part binding peptide with a diameter of ~40 nm (50). These caveospheres could be functionalized with an anti-CD4 antibody, and showed specific binding to CD4^+^ cells within a mixed PBMC population. Likewise, functionalization with a C-C chemokine receptor type 5 specific antibody resulted in specific binding and uptake by CD4^+^ T cells.

As an alternative approach for T cell targeting, we conjugated interleukin (IL-)2 to hydroxyethyl starch (HES-)NP (Ø ~215 nm) and showed that IL-2 retained its biological activity as tested in an IL-2 bioassay (51). Further, human CD4^+^ T cells polyclonally pre-activated to express the IL-2 receptor CD25 internalized IL-2-coated HES-NP, and showed a higher extent of proliferation, confirming that IL-2 in a dual manner served both as a targeting and costimulatory agent by addressing CD25. Efficient engagement was also observed for CD4^+^CD25^high^ Treg. CD25-dependent T cell binding was achieved as well in *in vivo* experiments using immunodeficient Rag2^–/–^γc^–/–^ mice that had been reconstituted with human CD4^+^ T cells.

## Reprogramming of Exhausted T Effector Cells and Treg

**Figure 2:**
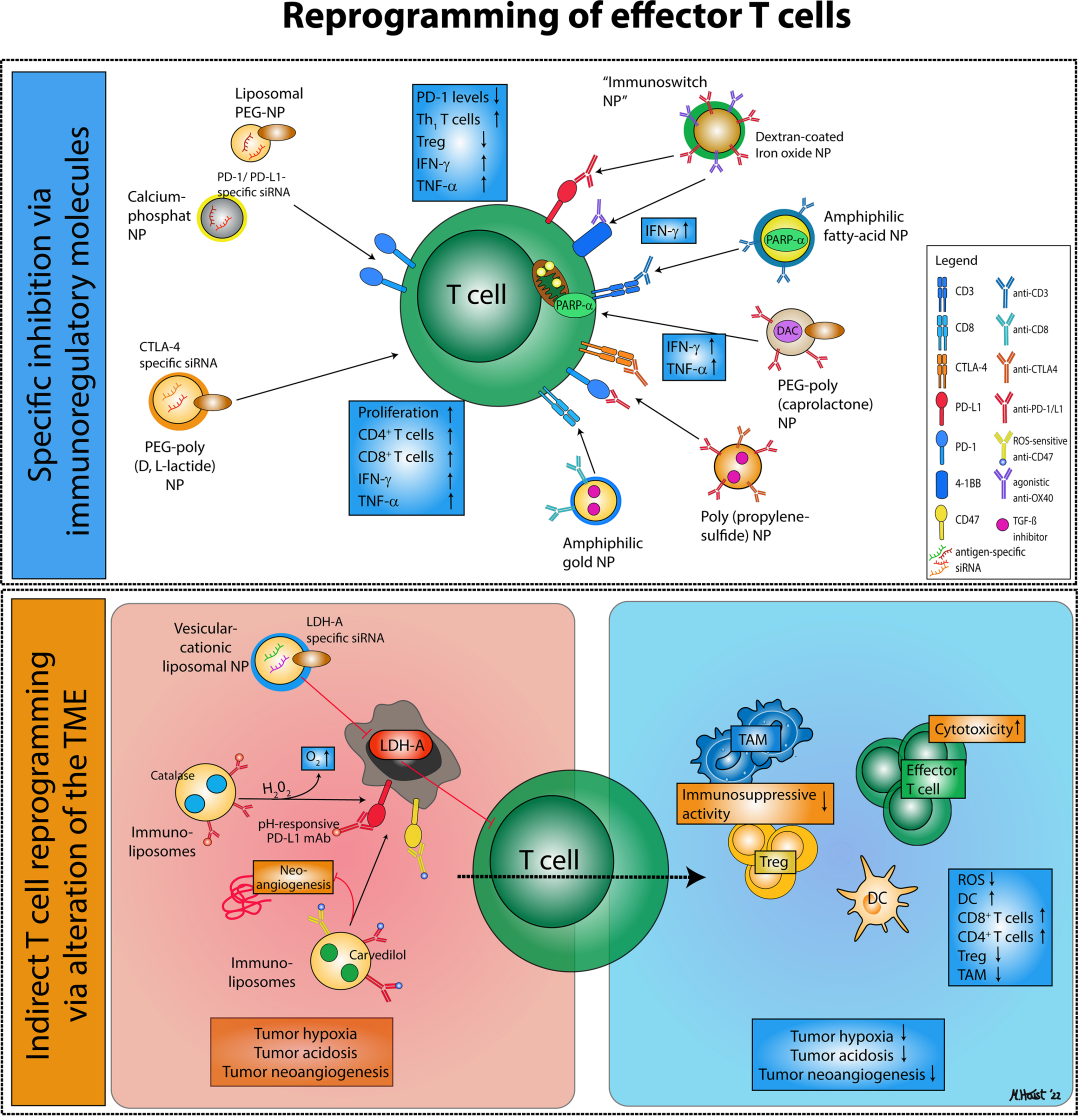
Nanodrug-based approaches for effector T cell reprogramming. Reprogramming of exhausted effector T cells and regulatory T cells *via* nanodrugs can be distinguished into two major categories: (1) Nanoparticles that specifically inhibit immunoregulatory molecules on effector T cells or regulatory T cells, thereby enhancing effector T cell activity. (2) Nanoparticles targeting immunosuppressive mechanisms characteristic of the tumor microenvironment that favor the accumulation of immunosuppressive cell types and an alleviate an effective anti-tumor immune response.

In the following, strategies that aimed to revert the exhausted state of T effector cells and to overcome the immunoregulatory function of Treg are discussed (**Figure 2**). In a proof-of-concept study focusing on NP-mediated RNA interference in Treg, laser-generated Au-based NP were coupled with a locked nucleic acid, that encompassed a green fluorescent protein (GFP)-specific siRNA, and a cell penetrating peptide to enhance endocytic particle internalization (52). This NP formulation (Ø ~6.8 nm) was internalized at high extent by splenic CD11b^+^ myeloid cell populations, and within the CD4^+^ T cell population by double as many CD4^+^CD25^+^ Treg (~ 10%) as CD4^+^CD25^-^ T cells. Incubation of Treg isolated from GFP-expressing reporter mice with GFP-siRNA coupled Au-NP resulted in a strong down-regulation of the GFP reporter on protein level after two days (52).

Another early study aimed to inhibit the expression of the immune checkpoint cytotoxic T lymphocyte-associated protein (CTLA-)4 in T cells by siRNA using NP composed of PEG-poly(D,L-lactide) and the cationic lipid N,N-bis(2-hydroxyethyl)-N-methyl-N-(2-cholesteryoxycarbonyl-aminoethyl) ammonium bromide as a carrier (Ø ~142 nm) (53). Treatment of antigen-specifically stimulated CD4^+^ T cells with CTLA-4 siRNA-delivering NP enhanced their proliferative activity. Systemic administration of these NP to melanoma-burdened mice resulted in strongly reduced tumor growth, which was accompanied which was accompanied by an overall increased tumor infiltration of CD4^+^ and CD8^+^ T effector cells, higher levels of T helper (Th)1/CTL-associated IFN-γ and tumor necrosis factor (TNF)-α in serum, whereas the number of Treg remained unaltered. T cell activation may also be extended by NP-aided inhibition of the inhibitory PD-1 signaling axis as demonstrated by Wu and coworkers by using calcium phosphate NP coated with (dioleoyl-3-trimethylammonium propane) DOTAP, dioleoylphosphatidylcholine and cholesterol, that encapsulated siRNA specific for PD-1 and PD ligand(L)1 (Ø ~30 nm) (54). These NP yielded downregulation of PD-1 expression in primary human tumor-infiltrating leukocytes (TIL) obtained from breast cancer specimen, and enhanced their tumor cell killing activity *in vitro*, accompanied by elevated IFN-γ and TNF-α contents. In a similar approach, liposomes composed of cholesterol, DOTAP, dioleoylphosphatidylethoanolamine (DOPE), and PEG were complexed with PD-1 specific siRNA (Ø ~87 nm), which engaged splenic CD8^+^ T cells *in vitro*, conferring downregulation of PD-1 expression (55). When systemically administered to melanoma-burdened mice, these liposomes enriched at the tumor site and in lymph nodes. By this approach the composition of tumor-infiltrating T cells was altered, displaying a higher frequency of Th1 cells on the expense of Treg and Th2 cells, and accompanied by lowered tumor growth.

Kosmides and co-workers aimed to enhance T cell effector activities by generating dextran-coated iron oxide NP that were conjugated with a PD-L1 blocking and an agonistic 4-1BB antibody (56). This so-called immunoswitch NP (Ø ~80 nm) was intended to target 4-1BB^+^ and PD-L1^+^ tumor cells. On the one hand this NP prevented binding of PD-L1 to PD-1 as expressed by T effector cells (57). On the other hand, the agonistic 4-1BB antibody engaged and triggered the costimulatory receptor 4-1BB on T cells (58). Thereby, the NP acted in a dual manner as a bridging and stimulatory agent. Immunoswitch NP enhanced the interaction of pre-activated murine CD8^+^ T cells with PD-L1 expressing melanoma, resulting in a stronger IFN-γ production and tumor cell killing *in vitro*. In a murine melanoma model, both intratumoral and intravenous injection of immunoswitch NP conferred strongly attenuated tumor growth, which was accompanied by higher frequencies of tumor-infiltrating IFN-γ^+^CD8^+^ T effector cells. In contrast, co-administration of soluble PD-L1 and 4-1BB antibodies was not effective.

Other studies aimed to inhibit the immunoregulatory effects of tumor growth factor (TFG-)β. In this regard, Yang and coworkers generated amphiphilic Au-NP that were loaded with a hydrophobic TGF-β inhibitor (SB525334) into the shell (Ø ~2.4 nm) (59). Additional conjugation with a CD8-specific antibody strongly enhanced the binding of this NP formulation to CD8^+^ T cells and at the same time attenuated its binding to other types of leukocytes *in vitro*. When applied systemically *in vivo*, besides CD8^+^ T cells also myeloid cell types, including conventional DC type 2, macrophages and granulocytic neutrophils engaged this NP at high-levels as well. Of note, also CD4^+^ T cells displayed considerable interaction with this NP formulation. Nonetheless, peripheral blood of tumor-burdened mice that were treated with this type of NP contained higher frequencies of CD8^+^TNF-α^+^IFN-γ^+^ T cells. However, tumor growth was not affected. In a similar approach Francis and coworkers generated poly(propylene sulfide) NP which upon conjugation of immune checkpoint PD-1- and CTLA-4- specific blocking antibodies, respectively, preferentially engaged CD4^+^ and CD8^+^ T cells in mice (60). In a melanoma model, co-administration of these NP formulations exerted similar inhibition of tumor growth as observed after the application of equimolar amounts of soluble antibodies applied in combination. Moreover, immune checkpoint-targeting NP loaded with a TGF-β inhibitor (SB-431542) conferred an even stronger tumor inhibition and prolonged the life span of melanoma-burdened mice.

Besides specific inhibition of distinct immunoregulatory molecules, another approach aimed to exploit that Treg within the TME depend on enhanced fatty acid oxidation (61). Muriski and coworkers generated fatty acid-conjugated quantum dots, which enabled quantification of fatty acid uptake by T cells (62). This quantum dot-derivative labeled tumor-derived CD4^+^Foxp3^+^ Treg at higher extent than any other tumor-infiltrating T cell population in a mouse model of intracranial tumors. In order to metabolically reprogram T cells within the TME, Kim and co-workers encapsulated the peroxisome proliferator-activated receptor (PARP-)α inducer fenofibrate in amphiphilic polygamma glutamic acid-based NP that were conjugated with a CD3-specific F(ab´)2 antibody fragment for pan T cell targeting (Ø ~150 nm) (63). This NP formulation enhanced the mitochondrial oxygen consumption rate of T cells under glucose-deficient conditions *via* PARP-α induced activation of fatty acid metabolism *in vitro*. In a murine melanoma model, intratumoral administration of this NP formulation resulted in higher frequencies of tumor-infiltrating T cells, elevated frequencies of IFN-γ and granzyme B expressing CD3^+^ T cells, and virtually abrogated tumor growth.

Based on the observation that the effector functions of tumor-infiltrating T effector cells are inhibited on genomic level by *de novo* gene promoter methylation (64), Hu and co-workers tested the efficacy of T cell-focused delivery of the DNA methyltransferase inhibitor 5-Aza-2´-deoxycytidine (DAC) (65). For this, PEG-poly(ε-caprolactone) NP were conjugated with the clinically employed PD-1 blocking antibody nivolumab to target PD-1 expressing T cells and were loaded with DAC. This formulation (Ø ~218 nm) enhanced IFN-γ production and cytotoxicity of PD-1 expressing CD8^+^ T cells in an *in vitro* assay, which suggested suitability for *in vivo* therapy.

In other approaches, enhanced tumor infiltration of T effector cells was not achieved by direct addressing, but by altering TME conditions. To overcome the acidic state of the TME, known to inhibit T effector cells (66), Zhang and coworkers aimed to inhibit lactate dehydrogenase A (LDHA) in the TME (67). For this, vesicular cationic lipid-assisted NP consisting of DOTAP and PEG-PLGA were loaded with Ldha-specific siRNA (Ø ∼95 nm). In a murine melanoma model, intraveneously administered NP accumulated intratumorally, and reduced Ldha expression, which elevated the pH value. Further, NP application decelerated tumor growth, which was further attenuated upon co-administration of a PD-1 blocking antibody. Tumor analysis revealed enhanced tumor infiltration of CD4^+^ and CD8^+^ T effector cells and lower numbers of CD4^+^Foxp3^+^ Treg. To overcome hypoxia, which constitutes another hallmark of solid tumors, immunoliposomes composed of lecithin, cholesterol and DSPE-PEG were loaded with Catalase to generate oxygen from break down of hydrogen peroxide (68). These NP were conjugated with a PD-L1 specific blocking antibody *via* a pH-responsive linker to target PD-L1 expressing tumor cells. In a murine melanoma model, immunoliposomes (Ø ∼118 nm) accumulated in the tumor, PD-L1 blocking antibody was released due to the acidic conditions, and the NP was internalized by tumor cells. Furthermore, the intratumoral hypoxic state was normalized, and a higher infiltration of the TME with CD8^+^ T cells, but not CD4^+^ T cells was observed. Treatment of tumor-burdened mice inhibited tumor growth at a higher extent as observed in response to the application of soluble PD-L1 blocking antibody, resulting in enhanced overall survival. In a subsequent study, the aforementioned immunoliposome platform was used to encapsulate carvedilol, intended to block neoangiogenesis within the tumor (69), and to conjugate CD47 and PD-L1 blocking antibodies *via* reactive oxygen species (ROS)-sensitive thioketal linkers (70). Functionalization with CD47-blocking antibody was dedicated to inhibit `don´t-eat-me´ signaling by tumor cells, which may express this receptor at high level (71). In melanoma-burdened mice intravenous administration of these multi-functionalized immunoliposomes (Ø ∼101 nm) attenuated ROS concentrations due to their reaction with the thioketal linkers (70). Moreover, TIL composition was strongly altered, characterized by attenuated levels of Treg and TAM and elevated frequencies of activated DC, M1-like macrophages and CD8^+^ T cells. Furthermore, blood vessel density was strongly reduced in the course of treatment, and the frequency of apoptotic tumor cells increased.

## CAR-T Cell Engineering

So far, CAR-T cells are generated *ex vivo* by transduction of patient-derived T cells with viral vectors encoding for the chimeric TCR, costimulatory moieties and eventually additional stimulatory factors (72). By now, CAR-T therapy has been approved by the FDA for treatment of lymphoma and leukemia (73). In order to overcome especially safety concerns associated with the use of viral entities, in a number of studies T cells have been transfected with mRNA by electroporation (74). However, also mRNA transfection of T cells by electroporation bears limitations with regard to method-associated cytotoxicity and low transfection efficacy. As an alternative especially lipid NP that complex mRNA and contain helper lipids for stabilization have been tested for *ex vivo* T cell transfection with mRNA (75–78) and plasmid DNA (79). Based on library screening, Billingsley and coworkers identified an ionizable lipid which induced comparable mRNA transfection efficiency at lower cytotoxicity on Jurkat T cells (75), and subsequently optimized this vector by varying the molar ratios of DOPE, cholesterol and lipid-anchored PEG (76). The optimized formulation conferred CAR mRNA transfection of primary T cells at similar efficacy as electroporation and viral transfer, yielding comparable CAR-T-mediated target cell killing. By screening a library encompassing chemically differing lipid-like formulations, termed lipidoids, Zhao and coworkers showed that lipidoids with an imidazole group yielded stronger reporter mRNA transfection efficacies of primary human CD8^+^ T cells *in vitro* (77), and was suitable for transfer of CAR-T mRNA into CD8^+^ T cells as reflected from potent killing activity of lymphoma cells (78). Furthermore, lipoids also mediated efficient *in vivo* transfection of T cells as assessed by applying Cre recombinase-encoding mRNA into a reporter mouse strain yielding dTomato expression in response to Cre recombinase-mediated recombination (77). Yu and coworkers screened a number of self-assembled NP composed of cyclodextrin-grafted branched PEI and adamantine-grafted polyamidoamine dendrimers for their suitability to mediate plasmid DNA transfection of human Jurkat T cells (79). Based on initial reporter mRNA transfection studies a formulation (Ø ~100 nm) was chosen and successfully evaluated for CAR plasmid DNA transfection and tumor cell killing.

NP have also been used to enable *in vivo* MRI of CAR-T cells: In two recent studies CAR-T cells were labeled with iron oxide-based spheric NP (Ø ~25 nm) (80) and nanoworms (Ø ~120 nm) (81). Luo and coworkers aimed to increase the stimulatory properties of CAR-T cells by binding interleukin-12 –coated human serum albumin-based NP onto the CAR-T cell surface by means of bioorthogenal chemistry (82). IL-12 was released in response to binding to tumor target cells, which resulted in an enhanced release of T cell-attracting chemokines and thereby increased CAR-T infiltration, yielding a positive feedback loop.

## Translation of Nano-Immunotherapy in Clinical Trials

In the past decade immune-oncology research has undergone substantial progress in understanding the regulatory mechanisms of anti-tumor immunity paving the way for novel immunotherapeutic agents such as ICI and CAR-T cells, which demonstrated impressive outcomes in various cancer types. In particular, the advent of ICI, that block PD-1, PD-L1 and CTLA-4, significantly improved treatment for a number of solid tumors, such as metastatic melanoma (83), non-small-cell lung cancer (84), merkel cell carcinoma (85), colon carcinoma (86), head-and-neck squamous cell carcinoma (HNSCC) (87) and others (88, 89). Also, CAR-T cell therapy showed remarkable long-term results in hematological diseases such as B cell malignancies (90–93). Still, a majority of patients may not derive significant benefit from immunotherapy treatment (35, 94), attributed to low immune cell infiltration into solid tumors, tumor antigen loss and the potent immunosuppressive character of the TME (95, 96).

In order to overcome treatment resistance and to enhance clinical responses to immunotherapy, new therapeutic strategies are needed that may synergize with current immunotherapeutic agents. In this context, nanodrugs are showing high potential in cancer immunotherapy as demonstrated in various preclinical trials as outlined above because they (1) allow for the selective targeted delivery of drugs, (2) may enhance immunogenic cell death (ICD), and (3) may reduce off-target toxicity (97). Also co-administration of several agents in a physically coupled form by one delivery platform is feasible with nanocarriers. Based on the promising results of preclinical trials a steadily increasing number of clinical studies are underway that investigate the clinical efficacy of T cell addressing nanodrugs. The number of these trials substantially increased after the approval of ICI and CAR-T cell therapies, which may be attributed to the potent synergistic effects hypothesized for T cell targeting nanodrugs and ICI treatments (98). Therefore, the majority of these studies are investigating on the combination of ICI and T cell addressing nanodrugs, whereas only a small number of trials examine the clinical efficacy of T cell addressing nanodrugs as monotherapy.

In the following, we will summarize and discuss the most important clinical trials on T cell-targeting nanodrugs for cancer immunotherapy. **Table 1** gives an overview of landmark clinical trials on T cell-targeting nanodrugs that mainly comprise indirect approaches to address T cells, such as DC-mediated T cell stimulation or immunomodulation of T cells as a bystander effect of an applied agent. Given the great number of clinical trials we will focus on the most advanced studies and further distinguish between nanodrugs directly addressing T cells and those indirectly affecting T cells. Among indirect T cell targeting nanodrugs, the majority of trials uses nano-formulated chemotherapeutics and mRNA-based nano-vaccines in combination with ICI therapies, which will be outlined in the following.

**Table 1:** Selection of clinical trials in cancer immunotherapy involving T cell targeting nanodrugs and biomaterials.

Formulation	Mechanism of action	Study	Study description	Arms and Interventions	Results	Ref
*Directly T cell addressing NP*						
*1. CAR-T cell therapy*						
anti-VEGFR2 CAR-T cells	Targeting VEGFR2 as tumor antigen	NCT01218867	Phase I/II trial: 24 patients with metastatic, refractory cancer	Lymphodepleting conditioning with cyclophosphamide, aldesleukin, and fludarabine, followed by different doses of anti-VEGFR2 CAR-T cells	Terminated due to PD in 23/24 patients and AE in 95.8% of patients	**-**
anti-GD2 CAR-T cells	Targeting GD2 as tumor antigen and including a suicide switch in case of toxicity (ICD9)	NCT02107963	Phase I trial: 15 patients with refractory GD2+ tumors	Lymphodepleting conditioning with cyclophosphamide, followed by different doses of anti-GD2 CAR-T cells; If unacceptable toxicity occurs AP1903 may be administered	Results pending	(99)
anti-GD2 CAR-T cells	Targeting GD2 as tumor antigen; C7R gene is added to increase the CAR-T cell survival	NCT03635632	Phase I trial: 94 patients with refractory or relapsed GD2+ solid cancers	Lymphodepletion with cyclophosphamide and fludarabine, followed by different doses of anti-C7R-GD2.CAR-T cells	still recruiting	**-**
anti-CD70 CAR-T cells	Targeting CD70 as tumor antigen	NCT02830724	Phase I/II trial: 124 patients with refractory or relapsed CD70 positive solid tumors	Lymphodepletion with cyclophosphamide fludarabine, and aldesleukin followed by different doses of anti-hCD70 CAR-T cells	still recruiting	–
B7-H3 targeting CAR-T cells	Targeting B7H3 as tumor antigen	NCT04483778	Phase I trial: 68 patients with relapsed or refractory B7H3 expressing advanced solid tumors	Arm A: Autologous T-cells genetically modified to express an B7H3-specific CAR Arm B: Autologous T-cells genetically modified to a bispecific B7H3xCD19 CAR	still recruiting	**-**
anti-gp100 CAR-T cells	Targeting gp100 as melanoma antigen	NCT03649529	Early phase I trial: 6 patients with relapsed or refractory gp100 positive melanoma	Patients undergo leukapheresis to isolate T cells; these will be modified and applied as GPA-TriMAR CAR-T cells	still recruiting	**-**
anti-NY-ESO-1 CAR-T cells	Targeting NY-ESO-1 as tumor antigen	NCT03638206	Phase I/II trial: 73 patients with refractory advanced cancer positive for NY-ESO-1, CD19 and other antigens	Different conditions depending on tumor entity. Following treatment with cyclophosphamide or fludarabine patients receive different CAR-T cells including anti-NY-ESO1	still recruiting	–
CD20 CAR-T cell	Targeting CD20 as melanoma antigen	NCT03893019	Early phase I trial: 15 patients with refractory, unresectable stage III or metastatic stage IV melanoma	Different doses of anti-CD20 CAR-T cells (MB-CART20.1)	still recruiting	–
anti-IL13Ra CAR-T cells	Targeting IL13Ra as melanoma antigen	NCT04119024	Phase I trial: 24 patients with refractory, unresectable stage III or metastatic stage IV melanoma with confirmed IL-13Ra expression	Lymphodepletion with cyclophosphamide and fludarabine phosphate, followed by treatment with recombinant Interleukin-2, and IL13Ralpha2-specific Hinge-optimized 4-1BB-co-stimulatory CAR/Truncated CD19-expressing T Cells	still recruiting	–
*2. Other*						
Lipid Nanoparticle encapsulating mRNA encoding OX40L, IL-23, and IL-36γ (mRNA-2752)	T cell activation and stimulation following ligation with OX40L and treatment with IL-23 and IL-36γ	NCT03739931	Phase I: 264 patients with refractory, advanced or metastatic disease	Arm A: intratumoral mRNA-2752 monotherapy; Arm B: intratumoral mRNA-2752 + durvalumab; Arm C: intratumoral mRNA-2752 alone or in combination with durvalumab	still recruiting, but preliminary results showing low efficacy with 1 partial response among 17 patients	(100)
Lipid nanoparticle encapsulating mRNA encoding OX40L (mRNA-2416)	Enhanced T cell activity, IFN-γ and TNF-α synthesis; upregulation of activation molecules CD25, 4-1BB, OX40	NCT03323398	Phase I/II trial: 117 patients with advanced, refractory cancer	Arm A: Intratumoral mRNA-2416 monotherapy Arm B: Intratumoral mRNA-2416 in combination with durvalumab	active, but not recruiting; results pending	–
Saline formulated mixture of 4 mRNA encoding GM-CSF + IFNa2b + IL-12 single chain + IL-15 (SAR441000)	Antigen-specific T cell expansion, increased infiltration by Granzyme B T cells, formation of immune memory, Interferon-γ induction	NCT03871348	Phase I trial: 231 patients with advanced anti-PD-1 naïve and refractory solid tumors	Different treatment arms including monotherapy with intratumoral SAR441000 and combination of cemiplimab and intratumoral SAR441000	still recruiting, but preliminary results showing no dose limiting toxicities, but anti-tumor activity in some patients	(101)
Liposomal nanodrug delivering MUC1 lipid BLP24 and Monophosphoryl Lipid A (Tecemotide; L-BLP25, StimuVax)	Induction of Th1 polarization and CD8 T cell responses	NCT01462513	Phase II trial: 122 patients with CRC after curative resection of hepatic metastasis	Arm A: L-BLP25 monotherapy Arm B: placebo	Failed to reach primary endpoints; Median PFS: 6.1 *vs* 11.4 months; median OS: 62.8 *vs* not reached; severe AE: 29.1% *vs* 26.2%	
Artificial antigen presenting cells (aAPC) to generate Melan-A/MART1 specific T cells	Using aAPC to generate melanoma-specific cytotoxic T cells upon leukapharesis	NCT00512889	Phase I trial: 9 patients with unresectable stage III or metastatic stage IV melanoma and MART-1/ Melan-A expression	Treatment either with aAPC generated MART-1/Melan-A specific CTL or treatment with combined aAPC-generated CTL plus GM-CSF and irradiation of cutaneous melanoma lesions	No results posted; aAPC educated CTL could survive for prolonged periods, trafficked to the tumor and established antitumor immunologic memory	(102)
aAPC to expand multiple antigen-specific T cells (MASE-T)	Using aAPCS scaffolds for antigen-driven T cell expansion and enrichment of T cells specific for melanoma antigens	NCT04904185	Phase I trial: 12 patients with ICB-refractory metastatic melanoma	Part A: Lymphodepletion with fludarabine and cyclophosphamide prior to ex-vivo, aAPC expanded T cell transfer; Part B: Lymphodepletion with fludarabine and cyclophosphamide prior to ex-vivo, aAPC expanded T cell transfer in combination with pembrolizumab	still recruiting	**-**
**Indirectly T cell targeting NP**						
*1. Protein based NP*						
Nanoformulation of paclitaxel which is incorporated in albumin nanoparticles as a carrier	Enhanced infiltration of CTL and DC and reduction of Treg numbers; contributes to TAM polarization towards M1 phenotype	NCT02425891	Phase III trial: 902 patients with unresected triple-negative breast cancer	Arm A: atezolizumab plus nab-paclitaxel Arm B: Placebo plus nab-paclitaxel	Median PFS: 7.2 *vs* 5.5 months, p=0.002; median OS: 21.3 *vs* 17.6 months, p=0.08; AE that led to treatment discontinuation: 15.9 *vs* 8.2%	(103, 104)
Nanoformulation of paclitaxel which is incorporated in albumin nanoparticles as a carrier	Enhanced infiltration of CTL and DC and reduction of Treg numbers; contributes to TAM polarization towards M1 phenotype	NCT02367781	Phase III trial: 723 patients with chemotherapy-naïve patients with stage IV NSCLC	Arm A: nab-paclitaxel plus carboplatin in combination with atezolizumab Arm B: nab-paclitaxel plus carboplatin	Median OS: 18.6 *vs* 13.9 months (p=0.033); median PFS: 7.0 *vs* 5.5 months (p<0.0001), serious TAE were reported in 24% *vs* 13% of patients	(105)
*2. Polymer-drug/protein conjugates*						
PEGylated liposomal formulation of doxorubicin	Induction of ICD, depletion of myeloid-derived suppressor cells; enhanced cancer-cell susceptibility to CTL-released granzyme B	NCT02580058	Phase III trial: 566 patients with platinum-resistant ovarian cancer	Arm A: avelumb in combination with Doxil Arm B: Doxil monotherapy Arm C: avelumab monotherapy	Median PFS: 3.7 *vs* 3.5 months (Arm A *vs* B p=0.03) *vs* 1.9 months (Arm C); median OS: 15.7 *vs* 13.1 months (p=0.021) *vs* 11.8 months; Serious TAE were reported in 18% *vs* 11% *vs* 7 %	(106)
Nanoliposomal PEGylated Irinotecan	Depletion of regulatory T cells and upregulation of MHC-I and PD-L1 expression, resulting in enhanced anti-tumor activity	NCT01494506	Phase III trial: 417 patients with gemcitabine-resistant, metastatic pancreatic cancer	Arm A: Nanoliposomal irinotecan Arm B: 5-FU plus leucovorin Arm C: Nanoliposomal irinotecan in combination with 5-FU and leucovorin	Median PFS: 3.1 *vs* 1.5 (p=0.0001) *vs* 1.6 months (p=0.1); Median OS: 6.1 *vs* 4.2 *vs* 4.9 months (Arm B *vs* C *vs* A; p=0.012); Serious TAE were reported in 48% *vs* 45% *vs* 61%	(107)
*3. Liposomal or lipid-based NP*						
Formulation of RNA-drug products against the melanoma antigens NY-ESO-1, tyrosinase, MAGE 3 and TPTE with liposomes that form RNA-lipoplexes (RNA-LPX; BNT-111)	DC maturation and activation, induction of antigen-specific T cell responses	NCT02410733	Phase I trial: 119 patients with advanced, refractory melanoma	Lipo-MERIT + nivolumab *vs* Lipo-MERIT monotherapy	still active but not recruiting; interim data: Monotherapy arm: ORR of 4/25 and DCR of 11/25; Combination therapy: ORR of 6/17	(108, 109)
mRNA-4157 encapsulated in lipids	DC maturation and activation, induction of antigen-specific T cell responses	NCT03313778	Phase I trial: 142 patients with locally advanced or metastatic solid malignancies including NSCLC, CRC, HNSCC, urothelial carcinoma or melanoma	Arm A: mRNA-4157 monotherapy Arm B: mRNA-4157 monotherapy in combination with pembrolizumab	still recruiting; interim analysis revealed no disease-limiting toxicities or serious TAE; Arm A: 11/13 remained disease free for median of 10 months Arm B: PR in 5/20 patients and SD in 6/20 patients	(110)
mRNA-4157 encapsulated in lipids	DC maturation and activation, induction of antigen-specific T cell responses	NCT03897881	Phase II trial: 157 patients with high-risk melanoma upon complete resection of lymph node metastasis	Arm A: mRNA-4157 in combination with pembrolizumab Arm B: pembrolizumab monotherapy	active, but interim results pending	–
Paclitaxel loaded lipid core NPs	DC maturation and T cell activation	–	Phase II trial: 14 patients with refractory ovarian carcinoma	Single-group arm treated with paclitaxel carried in non-protein lipid core nanoparticles (PTX-LDE)	Median PFS: 3.0 months with no major toxicities	(111, 112)
Eribulin encapsulated in liposomal formulation (E7389)	Vascular remodeling and facilitation of immune cell recruitment into the tumor	NCT04078295	Phase Ib/II trial: 116 patients with advanced, nonresectable or recurrent solid tumor	Arm A: E7389-LF in combination with nivolumab	active, but not recruiting; interim data: ORR was 17.6% and DCR was 79.4%; median PFS: 3.7 months; median OS: 7.6 months	(113)
STP705 Liposomal formulation of two siRNA oligonucleotides targeting TGF-ß1 and COX-2 mRNA	Increased T effector cell activation, cytokine secretion and proliferation	NCT04844983	Phase II trial: 100 patients with cutaneous squamous cell carcinoma *in-situ*	Arm A: Intralesional STP705 Arm B: Placebo Saline	still recruiting	–
Formulation of RNA-drug products against 5 antigens with liposomes that form RNA-lipoplexes (RNA-LPX; W_pro1 cancer vaccine)	DC maturation and activation, induction of antigen-specific T cell responses	NCT04382898	Phase I/II trial: 130 patients with metastatic castration-resistant prostate carcinoma	Arm A: W_pro1 in combination with cemiplimab Arm B: W_pro 1 monotherapy	still recruiting	(114)
Formulation of RNA-drug products against 3 antigens with liposomes that form RNA-lipoplexes (RNA-LPX; W_ova1 Vaccine)	DC activation and T cell stimulation	NCT04163094	Phase I trial: 10 patients with ovarian carcinoma eligible for neo-adjuvant chemotherapy	Single-treatment group: W_ova 1 monotherapy during neo-adjuvant chemotherapy and subsequent adjuvant chemotherapy with carboplatin/paclitaxel	still recruiting	–
Formulation of RNA-drug products against HPV 16 with liposomes that form RNA-lipoplexes (RNA-LPX; HPV16 E7; HARE40)	Priming and activation of effector and memory T cells	NCT03418480	Phase I/II trial: 44 patients with HPV16+, refractory HNSCC, anogenital, cervical or penile carcinoma	Arm A: HPV16 E7 RNA-LPX monotherapy Arm B: HPV16 E7 RNA-LPX in combination with anti-CD40	active, but not recruiting; interim data pending	(115)
IVAC_W_bre1_uID	DC activation and T cell stimulation	NCT02316457	Phase I trial: 42 patients with pT1, N0, M0 triple-negative breast cancer	Arm 1: IVAC_W_bre1_uiD monotherapy Arm 2: IVAC_W_bre1_uID/IVAC_M_uID personalized vaccine monotherapy Arm 3: IVAC_W_bre1_uID + RBLTet.1 with 3 variant RNA	active but not recruiting; interim data pending	(116)
Lipid-formulated mRNA with tumor and lysosome-associated membrane glycoprotein-fused cytomegalovirus pp65 mRNA	Induction of ICD, DC activation and T cell stimulation	NCT04573140	Phase I trial: 28 patients with newly diagnosed pediatric high-grade gliomas and adult glioblastoma	Single-treatment trial: RNA-loaded lipid particles with total tumor mRNA and pp65	still recruiting	–
Lipid-formulated mRNA encoding for different kRAS mutations (mRNA-5671/V941)	Induction of ICD, DC activation and T cell stimulation	NCT03948763	Phase I trial: 100 patients with KRAS-mutant advanced or metastatic NSCLC, CRC or pancreatic adenocarcinoma	Arm A: mRNA-5671 monotherapy Arm B: mRNA-5671 in combination with pembrolizumab	active, but results pending	–
Lipid-formulated NP with individualized tumor neoantigens (RNA-LPX; RO7198457)	DC activation and T cell stimulation	NCT03289962	Phase Ia/b trial: 272 patients with locally advanced or metastatic, refractory solid tumors	Arm A: RO7198457 monotherapy Arm B: RO7198457 in combination with atezolizumab	active, but not recruiting; interim data of 26 patients showed 1 CR and 11 patients with SD	(117)
Lipid-formulated NP with individualized tumor neoantigens (RNA-LPX; RO7198457)	DC activation and T cell stimulation	NCT03815058	Phase II trial: 131 patients with previously untreated advanced melanoma	Arm A: pembrolizumab monotherapy Arm B: RO7198457 in combination with pembrolizumab	active, but not recruiting, interim data pending	–
*4. Viral vectors*						
Imlygic in combination with pembrolizumab	Tumor lysis, DC activation; T cell stimulation and infiltration	NCT04068181	Phase II trial: 72 patients with stage IIIB-IV melanoma who have progressed on anti-PD-1 therapy	Single-arm treatment: intralesional Talimogene laherparepvec in combination with pembrolizumab	active, but results still pending	
RP1 oncolytic virus (HSV-1) that expresses a fusogenic glycoprotein (GALV-GP-R) and GM-CSF	Tumor lysis, DC activation; T cell stimulation and infiltration	NCT04050436	Phase II trial: 180 patients with advanced cutaneous squamous cell carcinoma of the skin	Arm A: cemiplimab in combination with RP1 Arm B: cemiplimab monotherapy	still recruiting	(118)
*5. Inorganic Nanoparticles*						
Hafnium oxide nanoparticles (NBXTR3)	Enhance ICD *via* electron production, T cell activation	NCT03589339 and NCT02379845	Phase I trial: 60 patients with locally advanced cancers treated with anti-PD-1 therapy and phase II/III trial with 180 patients with advanced soft-tissue sarcoma	Arm A: intratumoral injection of NBTXR3 followed by radiation and anti-PD-1 therapy with nivolumab or pembrolizumab Arm B: radiotherapy followed by anti-PD-1 monotherapy	Results from phase II/III trial: pCR in 16% *vs* 8% (p=0.044); serious AE 39% *vs* 30%	(119, 120)

aAPC, artificial antigen presenting cells; (p)CR, (pathological) complete response; CRC, colorectal carcinoma; CTL, cytotoxic T lymphocytes; DC, dendritic cells; DCR, disease-control rate; GD2, disialoganglioside 2; GM-CSF, granulocyte-monocyte colony stimulating factor; HNSCC, head-and-neck squamous cell carcinoma; HPV, human papillomavirus; HSV, herpes simplex virus; ICB, immune-checkpoint blockade; ICD, immunogenic cell death; IFN, interferon; NSCLC, non-small cell lung cancer; ORR, objective response rate; OS, overall survival; PD, progressive disease; PFS, progression-free survival; PR, partial response; SD, stable disease; (T)AE, (treatment-related) adverse events; TGF-ß, transforming growth factor beta; VEGFR, vascular endothelial growth factor receptor.

## Nanodrugs Addressing T Cells Indirectly

Nanodrugs modulating T cells encompass a wide spectrum of different anticancer modalities, that act (1) *via* induction of ICD, (2) DC-mediated T cell activation or (3) depletion/reprogramming of immunosuppressive cell types. In the following we will present three different types of such indirectly T cell modulating nanodrugs that include protein-based NP, polymer-drug conjugates and liposomal mRNA vaccines, as well as viral vectors.

## Protein-Based NP

The most common protein-based NP that affect T cells are nano-formulations of chemotherapeutic agents, which are incorporated in albumin-based NP as a carrier. In this regard, Abraxane was the first protein-based NP formulation that was approved by the FDA (121). Abraxane is a nano-formulation of the chemotherapeutic paclitaxel. Apart from direct tumoricidal effects of paclitaxel various immunomodulatory properties have been reported for this agent. In particular, paclitaxel was reported to enhance tumor infiltration of immune cells, such as lymphocytes and DC (111, 122). Also it has been shown that paclitaxel reduced the number of Tregs (123) and contributed to the repolarization of TAM towards an inflammatory M1 macrophage phenotype (124). More importantly, application of Abraxane resulted in higher response rates and substantially decreased side effects as compared to conventional paclitaxel treatment, which led to FDA approval for treatment of metastatic breast cancer, metastatic non-small cell lung cancer (NSCLC) and metastatic pancreatic cancer (125). Most clinical trials investigating the efficacy of Abraxane have been conducted in combination with other immunotherapy agents, in particular with PD-1/PD-L1 blocking antibodies (e.g. atezolizumab). Here, the IMpassion130 trial showed that Abraxane in combination with the PD-L1 inhibitor atezolizumab significantly improved the median progression-free survival (PFS) and overall survival (OS) of patients with advanced triple negative breast cancer (TNBC) as compared to Abraxane plus placebo, without inducing further adverse events (126). The combination of Abraxane and atezolizumab also demonstrated improved clinical efficacy when compared to anti-PD-1/PD-L1 antibodies, such as pembrolizumab or avelumab (127, 128). Therefore, Abraxane plus atezolizumab was the first immunotherapeutic regimen that obtained FDA approval for advanced TNBC, and later Abraxane, atezolizumab and carboplatin obtained also approval for metastatic non-squamous and squamous NSCLC (105, 129). Next to the improved delivery of Abraxane, the reduced toxicities, which avoided premedication with immunosuppressive corticosteroids, are considered particularly important for the clinical efficacy of the combination of Abraxane and PD-(L1) inhibitors and contribute to the immunomodulatory effects of Abraxane on T effector cells (130).

## Polymer-Drug/Protein Conjugates

Polymer-protein conjugates that are applied in cancer treatment are mainly PEGylated proteins as PEGylation effectively protects proteins from degradation and improves drug distribution (131). The PEGylated liposomal formulation of doxorubicin, Doxil, was the first nano-medicine approved by the FDA for ovarian cancer (OC), acquired immune deficiency syndrome (AIDS)-related Kaposi´s sarcoma and multiple myeloma (98). Apart from the direct tumoricidal effects of Doxil, it also displayed various immunomodulatory properties, such as the induction of ICD, the depletion of MDSC (132), and enhanced susceptibility of cancer cells to CTL-released granzyme B (133), all contributing to an elevated anti-tumor immune response (134, 135). Doxil enhanced these immunomodulatory properties as opposed to the overall immunosuppressive nature of free doxorubicin, which was also related to decreased off-target toxicities of the former (136). In contrast to doxorubicin, which has numerous toxicities including a cumulative dose-depending cardiotoxicity, its liposomal formulation (Doxil) showed effectively reduced cardiotoxicity and avoided premedication with corticosteroids (137). Most clinical trials evaluating the clinical efficacy of Doxil were conducted in combination with PD-1/PD-L1 targeting ICI. In the most advanced phase III JAVELIN Ovarian 2000 trial, which compared the combination of avelumab and Doxil and either agent alone in platinum-resistant OC patients, it has however been found that the combination of both agents did not significantly improve PFS or OS (106). Notably, the best response was observed for patients with PD-L1 positive tumors, indicating that the clinical benefit of combined ICI and nanodrug treatment may also be achieved by adequate biomarker stratification of patients. Also, Doxil has been tested in combination with various other ICI agents, such as pembrolizumab for breast cancer (138) or platinum-resistant OC (139), and showed similar or improved efficacy as compared to doxorubicin at reduced systemic toxicity. In order to better understand the role of biomarker stratification and ICD to improve overall ICI efficacy, a number of ongoing clinical trials investigate the combination of immunogenic chemotherapy with Doxil in combination with ICI such as atezolizumab, and the combination of ipilimumab and nivolumab in metastatic TNBC (NCT03164993 and NCT03409198) (140, 141).

In addition to the aforementioned trials Doxil has also been tested in combination with cytokines such as IL-12 in AIDS-related Kaposi´s sarcoma, and this regimen yielded rapid and sustained tumor responses (NCT00020449) (142). By contrast, in platinum-resistant OC the combination of IL-18 and Doxil showed no significantly improved response and survival data (143), highlighting the overall observation that the combination of Doxil and immunotherapy did not enhance clinical efficacy for all cancer patients, but a certain subgroup of patients only.

## Liposomal and Lipid-Based NP

Cancer vaccines may consist of mRNA that encodes for tumor-associated antigens, which after internalization by APC are translated, processed and presented to T cells, thus initiating tumor antigen-specific T cell responses (144). Despite promising results many earlier clinical trials investigating on cancer vaccines have failed due to poor cell entry of mRNA vaccines and enzymatic degradation of the cargo (145). Thus, nanocarriers developed to deliver mRNA vaccines to the site of interest without the risk of degradation are of special interest to cancer immunotherapy and liposomal formulations of mRNA vaccines have proven to be particularly efficient in stimulating DC-mediated T cell activity (98, 146).

One of the best known liposomal formulations of mRNA cancer vaccines has previously been tested in the Lipo-MERIT trial (NCT02410733) and demonstrated durable objective responses in ICI-experienced patients with unresectable stage III or stage IV melanoma (108, 109). Here, the nano vaccine is composed of a mixture of mRNA species encoding the melanoma tumor antigens New York esophageal squamous cell carcinoma (NY-ESO-)1, melanoma-associated antigen (MAGE-)3, tyrosinase and putative tyrosine-protein phosphatase (TPTE), and cationic liposomes, which upon intravenous injection effectively transfected APC such as DC and macrophages, thereby inducing strong tumor antigen-specific CD4^+^ and CD8^+^ T cell immunity (109). Due to the promising results observed in the Lipo-MERIT trial, further mRNA-based nano vaccines encoding for tumor-associated antigens (TAA) using a similar liposomal formulation have been initiated for patients with advanced prostate cancer (NCT04382898) (114), advanced HNSCC (NCT04534205), and advanced OC. In accordance, the phase I dose escalation study of mRNA-4157 in combination with pembrolizumab reported safety and efficacy in patients with resected solid tumors, including melanoma, NSCLC, colon carcinoma and bladder carcinoma (NCT03313778). Hence, a phase II study investigating on adjuvant treatment mRNA-4157 in patients with high-risk melanoma has been initiated recently (NCT03897881).

Immunization with the tumor-specific MAGE-3 antigen has been shown to induce robust immune responses for patients with metastatic melanoma (147), albeit in a phase III trial the immunostimulatory lipid AS15 failed to improve PFS as an adjuvant treatment strategy in patients with MAGE-A3 positive NSCLC (148). Also, liposomal DepoVax-0907 (DPX) that delivered seven peptide TAA increased persistent antigen-specific T cell responses in 39% of patients with breast, ovarian or prostate cancer (149). In the following trial the liposomal DPX platform using survivin class I peptides (DPX-Survivac) was applied in combination with immunomodulatory cyclophosphamide in patients with advanced OC and all patients receiving combination therapy presented with an antigen-specific immune response, which has also been attributed to the Treg depleting effects of cyclophosphamide (150). Due to the strong immune response observed in this trial, DPX-Survivac and cyclophosphamide are currently investigated in a phase II trial in combination with pembrolizumab for patients with recurrent diffuse large B cell lymphoma (NCT03349450) (151).

## Viral Vectors

T-cell targeting nanodrugs also comprise viral vectors that encode tumor proteins, cytokines and enzymes aiming to improve anti-cancer efficacy. In this regard, FDA-approved intralesional treatment of patients with unresectable melanoma and cutaneous or lymph node metastasis with Talimogen laherparepvec (T-VEC), an oncolytic herpes-simples type 1 virus encoding for granulocyte-macrophage colony-stimulating factor (GM-CSF), has demonstrated to enhance immune response to tumor antigens released after virus replication (152). While efficacy has been demonstrated mainly on the injected lesion, in some patients an abscopal systemic effect was also evident in distant organ metastasis, which has been attributed to the recruitment and activation of CTL (153). However, the overall response to T-VEC treatment was relatively low with an overall response rate of 26%, although the limited toxicity profile and survival rates prompted follow-up trials for metastatic melanoma and advanced cutaneous squamous cell carcinoma (NCT04050436) (118, 154–156).

## Direct T Cell Targeting Nanodrugs

As mentioned above, in contrast to indirectly T cell addressing agents, nanodrugs that directly address T cells have not widely progressed into clinical trials so far. The most prominent clinical trials involve CAR-T cell therapies, which have shown high efficacy in hematological malignancies (73), but could not reproduce these effects in many solid tumors as outlined in the following. Additionally, T cell addressing mRNA-based nanodrugs are increasingly moving into clinical trials and will therefore also be addressed in this chapter.

## CAR-T Cell Therapies for Solid Tumors

A number of clinical trials that assess the efficacy and safety of CAR-T cell therapy for solid tumors including melanoma are currently underway. As melanoma constitutes a model tumor for immunotherapy, we will in the following focus on CAR-T cell therapies for this deadliest type of skin cancers (see **Table 1**).

Only two studies are completed by now, and the only published results came from a single study conducted on 24 patients (results available in NCT01218867). Here, the safety and efficacy of anti- vascular endothelial growth factor receptor CAR-T cells subsequent to lymphodepleting conditioning with cyclophosphamide, Aldesleukin, fludarabine and IL-2 was investigated. As opposed to previous pre-clinical studies in B16 melanoma-bearing mice (157), this study was terminated due to lack in efficacy with 95.8% showing progressive disease and high rates of serious adverse events (95.8%), that mainly involved elevated liver enzymes. The other clinical trial that has been completed by now is a phase I trial on the 3^rd^ generation of anti-disialoganglioside (GD)2 CAR-T cells that have been tested in combination with pembrolizumab after prior lymphodepleting conditioning with cyclophosphamide in young adults with GD2 expressing solid tumors, such as sarcoma, neuroblastoma and melanoma (NCT02107963). GD2 is expressed specifically on tumors of neuroectodermal origin such as melanoma and neuroblastoma (158). CAR-T cells specific for GD2 have previously been reported to show potent effector functions without evidence of functional exhaustion, which persisted in patients treated within the phase I trial and could be augmented with additional anti-PD-1 combination therapy (99). In addition, another phase I trial using anti-GD2-C7R CAR-T cells after prior lymphodepleting conditioning with cyclophosphamide and fludarabine is currently underway for patients with GD2-positive tumors such as uveal melanoma (GAIL-N; NCT03635632). Moreover, a more recent phase I clinical trial is investigating the safety and efficacy of anti-B7H3 CAR-T cells and bispecific anti-B7H3xCD19 CAR-T cells in recurrent solid tumors including melanoma (NCT04483778). Further, phase I clinical trials that evaluate the efficacy of various CAR-T cell therapies for metastatic melanoma are summarized in **Table 1** and include CAR-T cells specific for the tumor antigens gp100, NY-ESO1, CD20, CD70 and IL-13a2.

Despite the success of CAR-T cells in the treatment of hematological cancers, several challenges still limit the efficacy of this approach in solid tumors, such as antigen selection, antigen loss and heterogeneity, the immunosuppressive TME, the lack of T cell infiltration into the tumor and off-target toxicity. In order to increase the efficacy of CAR-T cell therapy, a combinational approach has been proposed to overcome some of these challenges and indeed combination treatments with ICI or oncolytic viruses have previously been reported to augment overall efficacy in preclinical tumor models (159–161). Despite the potential synergistic effects of ICI and CAR-T cell therapy there are yet no clinical trials that assess the efficacy and safety of this combinational approach for advanced melanoma, but only in other solid tumors (NCT03726515).

## Other T Cell Targeting Nanoimmunotherapeutics

Next to intravenously administered mRNA encoding nano-vaccines that act *via* DC-mediated T cell activation, some clinical trials investigate the efficacy of intratumorally administered mRNA nanodrugs that directly address T cells. Here, the mRNA nanovaccine-2752, which encodes for human TNF superfamily member 4, IL-23 and IL-36γ, administered in combination with the PD-1 inhibitor durvalumab is currently under investigation in a phase I trial in patients suffering from advanced solid tumors such as melanoma, NSCLC, HNSCC and TNBC (NCT03739931). Preliminary data showed less promising results with only 1/17 patients presenting with a partial response upon intratumoral injection of mRNA-2752 (100). Another phase I clinical trial aimed to investigate the safety and efficacy of intratumorally applied SAR441000, delivering mRNA species that encode IL-12, interferon-α, GM-CSF, and IL-15, in combination with the PD-1 blocking antibody cemiplimab for patients with melanoma, breast cancer and other skin cancers (NCT03871348). Here, tumor responses have been observed more frequently, and were accompanied by increased T cell infiltration and antitumor activity even beyond the site of injection (101).

Additionally, Tecemotide, (L-BLP25, StimuVax), a liposomal nanodrug delivering mucin1 glycoprotein and the TLR4 agonist monophosphoryl lipid A (MPL) using the immunostimulatory lipid BLP24 has been tested in several clinical trials. MPL has been reported to induce Th1 polarization and CTL responses (162, 163). Despite these promising preclinical data and an acceptable safety profile in clinical trials, phase II and III trials failed to demonstrate a significant survival benefit for patients with NSCLC (164), but proved beneficial when applied in combination with cyclophosphamide in patients with stage IIIB locoregional disease (162). The subsequent trial investigating Tecemotide versus placebo in patients with colorectal cancer (CRC) after resection of liver metastases (NCT01462513) also failed to reach its primary endpoints as the median PFS (median PFS: 6.1 months *vs*. 11.4 months; p=0.18) and 3-year OS rates (3-year OS rates: 69.1 *vs* 79.1%; p=0.21) were non-significantly shorter in Tecemotide-treated patients as compared to the placebo control group (165).

Last, there are a number of clinical trials which have investigated the application of aAPC for tumor therapy. Here, it has been shown that aAPC were able to educate anti-tumor CTL to acquire a central memory and effector memory phenotype, resulting in T cell trafficking to the tumor site, and mediating clinical responses in advanced melanoma patients (NCT00512889) (102). In the ongoing phase I ImmPACT trial it will be investigated whether aAPC alone or in combination with pembrolizumab may expand antigen-specific endogenous T cells that may result in better objective responses and PFS for patients with metastatic melanoma upon T cell infusion and subsequent lymphocyte depleting chemotherapy with cyclophosphamide and fludarabine (NCT04904185).

## Conclusion and Perspective

The advent of ICI treatment has substantially improved the treatment landscape of many solid tumor types (83). However, innate and acquired resistance to these immunotherapies is frequently observed as solid tumors present with resistance mechanisms, such as an immunosuppressive TME and antigen loss, that impair clinical efficacy of currently available immunotherapies. To address these mechanisms of tumor immune evasion, the clinical application of T cell addressing nanodrugs is a promising approach as it may enhance the efficacy of other immunotherapeutics such as ICI, while reducing off-target toxicities.

In particular, it has been shown that local tumor responsiveness to ICI can be improved by codelivery with of adjuvants within the same nanocarrier or a combination of ICI with nano-formulated immune-modulatory agents. For example, coencapsulation of a PD-L1 blocking antibody and an inhibitor of indoleamine 2,3-dioxygenase 1 in a ROS-responsive polypeptide gel enabled the specific release of the cargo within the TME, characterized by at low pH and high ROS levels, subsequently reducing the local ROS level and enhancing anti-melanoma efficacy *in vivo* (166). Also, the coadministration of stimulator of IFN genes (STING) agonist-loaded poly(beta-amino-ester) NP with PD-1 blocking antibodies yielded strongly improved anti-tumor effects in established B16 melanomas-burdened mice as compared to either monotherapy and using STING agonist applied in its soluble form (167). Similarly, complexation of immunostimulatory CpG-rich DNA oligo (TLR9 ligand) with a nano-carrier carrying tumor-binding peptides enhanced NP accumulation within the tumor and improved responsiveness to coapplied CTLA-4 blocking antibodies (168).

Moreover, it has been found that nano-formulated TAA-specific mRNA vaccines (RNA-LPX) may induce potent anti-tumor T cell responses and enhance the efficacy of PD-(L)1 blocking antibodies and CAR-T cells (169) *via* selective delivery of RNA-LPX to APC in mice (170) and patients (171). These studies demonstrate the therapeutic potential of neoantigen-specific anti-tumor vaccines both when applied as a monotherapy as well as in combination with ICI. More recently, the same group also showed that the therapeutic benefit of RNA-LPX vaccination was preserved when employing non-mutant TAA that are primarily expressed by melanoma cells instead of neoantigens, and that this therapeutic approach may help to overcome ICI resistance (108). Next to the application of ICI in combination with other nano-formulated immune-modulatory agents, it has also been shown that nano-carriers may enhance the tumor-specific activity of ICI *per se* due to selective targeting of tumor-specific surface receptors, thereby additionally reducing off-target toxicities. In this regard, Ishihara and coworkers demonstrated that the conjugation of ICI (anti-CTLA4 and anti-PD-L1 mAb) to a matrix-binding peptide enhanced tissue retention of ICI, delayed tumor growth and prolonged survival in a B16-melanoma model while reducing systemic side effects (172). Finally, nanodrugs may also serve to overcome ICI resistance by altering hostile TME conditions. More specifically, it has been shown that TME-targeted nanoparticles can (a) overcome the physico-chemical barriers (ECM) of the TME (173), the acidic (67) and hypoxic (174) metabolic state of the TME, and (b) modulate immunosuppressive cells, such as Tregs, TAM or MDSC that frequently restrain therapy response in cancer immunotherapy (175). For example, Ou and coworkers demonstrated in a B16 melanoma model a synergistic anti-tumor effect of a Treg-specific tLyp1 peptide-conjugated and Imatinib-loaded NP in combination with an anti-CTLA4 antibody, that has been attributed to the downregulation of immunosuppressive Treg and an enhanced activation of CD8^+^ T cells (176). The tLyp peptide is a cell-penetrating peptide that has a high affinity for the Neuopilin-1 (Nrp1) surface receptor, which is expressed by many cancer types and by the majority of Treg (175). While the aforementioned combinational regimens may help to overcome ICI resistance in general, it will however be necessary to identify the individual resistance mechanisms that are present in each patient as a prerequisite to target these with personalized combination therapies of T cell-addressing nanodrugs and other immunotherapeutics in order to derive the maximal clinical benefit of specific nanodrug-combination regimens. Therefore, the role of immune biomarkers in clinical immune-oncology will also be critical with regard to T cell addressing nanodrugs (98, 177).

In general, the design the nanodrugs will need to take into account the potential of unwanted cellular interactions *in vivo*, which may not be recognized in standard *in vitro* assays when testing NP interaction with the intended target cell type only. In this regard, we have recently shown that non-directed conjugation of antibodies to the NP surface for cell type-specific targeting promoted strong liver accumulation of the NP due to binding of the exposed constant Fc part to Fc receptors expressed by liver sinusoidal endothelial cells (LSEC) at high density (178). Besides LSEC, many other immune cell types also express Fc receptors, which in general serve to recognize antibody-opsonized pathogens (179) and immune complexes (180). Such unwanted effects can be minimized e.g. by either Fc-directed antibody conjugation (181) and by antibody fragments devoid of the Fc part (182). Furthermore, NP may adsorb serum factors *in vivo* which in turn may alter their biodistribution and cellular interaction as shown by us and others (183, 184). The composition of this so-called protein corona depends on the surface characteristics of the nanodrug like charge and hydrophobicity/hydrophilicity. Besides passive adsorption of serum factors, the NP surface may also trigger the innate immune system as shown by us and others for NP coated with lectin like dextran and starch to achieve biocompatibility that triggered lectin-dependent complement activation (103, 184). This resulted in opsonization of the NP with active complement C3, which in turn yielded strong binding to B cells (103) and myeloid cell types (184) *via* their complement receptors and subsequent internalization. The formation of a protein corona and accordingly uptake by unwanted cell types can be strongly reduced by conjugation with PEG e.g. in a brush-like conformation at high density as recently shown by us (185) or by using materials that scarcely bind serum factors as e.g. polysarcosin (178, 186).

## Author Contributions

MH, VM, and MB wrote the manuscript. MH prepared the figures and table. All authors contributed to the article and approved the submitted version.

## Funding

This work was supported by the Deutsche Forschungsgemeinschaft (DFG, German Research Foundation) (SFB1066, Q2, Q6N, B11 [VM] and B15N [MB], and a Walter-Benjamin Fellowship (project number: 507666201) [MH]), and the University Medical Center Mainz (intramural grants: MH, MB; Research Center for Immunotherapy [FZI]: VM, MB).

## Conflict of Interest

The authors declare that the research was conducted in the absence of any commercial or financial relationships that could be construed as a potential conflict of interest.

## Publisher’s Note

All claims expressed in this article are solely those of the authors and do not necessarily represent those of their affiliated organizations, or those of the publisher, the editors and the reviewers. Any product that may be evaluated in this article, or claim that may be made by its manufacturer, is not guaranteed or endorsed by the publisher.

## References

[1] Xiang J Zhao R Wang B Sun X Guo X Tan S. Advanced Nano-Carriers for Anti-Tumor Drug Loading. Front Oncol (2021) 11:758143. doi: 10.3389/fonc.2021.758143 34604097 PMC8481913

[2] Chen F Wang Y Gao J Saeed M Li T Wang W. Nanobiomaterial-Based Vaccination Immunotherapy of Cancer. Biomaterials (2021) 270:120709. doi: 10.1016/j.biomaterials.2021.120709 33581608

[3] Germic N Frangez Z Yousefi S Simon HU. Regulation of the Innate Immune System by Autophagy: Monocytes, Macrophages, Dendritic Cells and Antigen Presentation. Cell Death Differ (2019) 26(4):715–27. doi: 10.1038/s41418-019-0297-6 PMC6460400 30737475

[4] Dowling JK Mansell A. Toll-Like Receptors: The Swiss Army Knife of Immunity and Vaccine Development. Clin Transl Immunol (2016) 5(5):e85. doi: 10.1038/cti.2016.22 PMC4910119 27350884

[5] Haist M Stege H Grabbe S Bros M. The Functional Crosstalk Between Myeloid-Derived Suppressor Cells and Regulatory T Cells Within the Immunosuppressive Tumor Microenvironment. Cancers (Basel) (2021) 13(2):3. doi: 10.3390/cancers13020210 PMC7827203 33430105

[6] Groth C Weber R Lasser S Özbay FG Kurzay A Petrova V. Tumor Promoting Capacity of Polymorphonuclear Myeloid-Derived Suppressor Cells and Their Neutralization. Int J Cancer (2021) 149(9):1628–38. doi: 10.1002/ijc.33731 34224592

[7] Yano H Andrews LP Workman CJ Vignali DAA. Intratumoral Regulatory T Cells: Markers, Subsets and Their Impact on Anti-Tumor Immunity. Immunology (2019) 157(3):232–47. doi: 10.1111/imm.13067 PMC6587321 31087644

[8] Peng SY Chen L Deng RH Li H Liu XH Zheng DW. Non-Depleting Reformation of Immunosuppressive Myeloid Cells to Broaden the Application of Anti-PD Therapy. Nanoscale (2021) 13(8):4420–31. doi: 10.1039/D1NR00830G 33616147

[9] Rhodes KR Green JJ. Nanoscale Artificial Antigen Presenting Cells for Cancer Immunotherapy. Mol Immunol (2018) 98:13–8. doi: 10.1016/j.molimm.2018.02.016 PMC6084459 29525074

[10] Matic J Deeg J Scheffold A Goldstein I Spatz JP. Fine Tuning and Efficient T Cell Activation With Stimulatory Acd3 Nanoarrays. Nano Lett (2013) 13(11):5090–7. doi: 10.1021/nl4022623 PMC3834297 24111628

[11] Est-Witte SE Livingston NK Omotoso MO Green JJ Schneck JP. Nanoparticles for Generating Antigen-Specific T Cells for Immunotherapy. Semin Immunol (2021) 56:101541. doi: 10.1016/j.smim.2021.101541 34922816 PMC8900015

[12] Lambert LH Goebrecht GK De Leo SE O'Connor RS Nunez-Cruz S Li TD. Improving T Cell Expansion With a Soft Touch. Nano Lett (2017) 17(2):821–6. doi: 10.1021/acs.nanolett.6b04071 PMC5504474 28122453

[13] Guasch J Muth CA Diemer J Riahinezhad H Spatz JP. Integrin-Assisted T-Cell Activation on Nanostructured Hydrogels. Nano Lett (2017) 17(10):6110–6. doi: 10.1021/acs.nanolett.7b02636 28876947

[14] Bednarczyk M Stege H Grabbe S Bros M. β2 Integrins-Multi-Functional Leukocyte Receptors in Health and Disease. Int J Mol Sci (2020) 21(4):8. doi: 10.3390/ijms21041402 PMC7073085 32092981

[15] Wu PH Opadele AE Onodera Y Nam JM. Targeting Integrins in Cancer Nanomedicine: Applications in Cancer Diagnosis and Therapy. Cancers (Basel) (2019) 11(11):4. doi: 10.3390/cancers11111783 PMC6895796 31766201

[16] Guasch J Hoffmann M Diemer J Riahinezhad H Neubauer S Kessler H. Combining Adhesive Nanostructured Surfaces and Costimulatory Signals to Increase T Cell Activation. Nano Lett (2018) 18(9):5899–904. doi: 10.1021/acs.nanolett.8b02588 30088769

[17] Chatila T Silverman L Miller R Geha R. Mechanisms of T Cell Activation by the Calcium Ionophore Ionomycin. J Immunol (1989) 143(4):1283–9. 2545785

[18] Hammink R Weiden J Voerman D Popelier C Eggermont LJ Schluck M. Semiflexible Immunobrushes Induce Enhanced T Cell Activation and Expansion. ACS Appl Mater Interf (2021) 13(14):16007–18. doi: 10.1021/acsami.0c21994 PMC8045021 33797875

[19] Lee K Yu Y. Janus Nanoparticles for T Cell Activation: Clustering Ligands to Enhance Stimulation. J Mater Chem B (2017) 5(23):4410–5. doi: 10.1039/C7TB00150A PMC5617359 28966791

[20] Vis B Hewitt RE Monie TP Fairbairn C Turner SD Kinrade SD. Ultrasmall Silica Nanoparticles Directly Ligate the T Cell Receptor Complex. Proc Natl Acad Sci USA (2020) 117(1):285–91. doi: 10.1073/pnas.1911360117 PMC6955355 31871161

[21] Ceuppens JL Baroja ML Lorre K Van Damme J Billiau A. Human T Cell Activation With Phytohemagglutinin. The Function of IL-6 as an Accessory Signal. J Immunol (1988) 141(11):3868–74. 3263438

[22] He S Simpson BK Sun H Ngadi MO Ma Y Huang T. Phaseolus Vulgaris Lectins: A Systematic Review of Characteristics and Health Implications. Crit Rev Food Sci Nutr (2018) 58(1):70–83. doi: 10.1080/10408398.2015.1096234 26479307

[23] Alhallak K Sun J Muz B Jeske A O'Neal J Ritchey JK. Liposomal Phytohemagglutinin: *In Vivo* T-Cell Activator as a Novel Pan-Cancer Immunotherapy. J Cell Mol Med (2022) 26(3):940–4. doi: 10.1111/jcmm.16885 PMC8817130 35014164

[24] Zupke O Distler E Jürchott A Paiphansiri U Dass M Thomas S. Nanoparticles and Antigen-Specific T-Cell Therapeutics: A Comprehensive Study on Uptake and Release. Nanomed (Lond) (2015) 10(7):1063–76. doi: 10.2217/nnm.14.160 25929565

[25] Liu L Ye Q Wu Y Hsieh WY Chen CL Shen HH. Tracking T-Cells *In Vivo* With a New Nano-Sized MRI Contrast Agent. Nanomedicine (2012) 8(8):1345–54. doi: 10.1016/j.nano.2012.02.017 PMC3383940 22406186

[26] Mühlberger M Unterweger H Band J Lehmann C Heger L Dudziak D. Loading of Primary Human T Lymphocytes With Citrate-Coated Superparamagnetic Iron Oxide Nanoparticles Does Not Impair Their Activation After Polyclonal Stimulation. Cells (2020) 9(2):2. doi: 10.3390/cells9020342 PMC7072432 32024193

[27] Boosz P Pfister F Stein R Friedrich B Fester L Band J. Citrate-Coated Superparamagnetic Iron Oxide Nanoparticles Enable a Stable Non-Spilling Loading of T Cells and Their Magnetic Accumulation. Cancers (Basel) (2021) 13(16):2. doi: 10.3390/cancers13164143 PMC8394404 34439296

[28] Huq R Samuel EL Sikkema WK Nilewski LG Lee T Tanner MR. Preferential Uptake of Antioxidant Carbon Nanoparticles by T Lymphocytes for Immunomodulation. Sci Rep (2016) 6:33808. doi: 10.1038/srep33808 27654170 PMC5031970

[29] Jalilov AS Zhang C Samuel EL Sikkema WK Wu G Berka V. Mechanistic Study of the Conversion of Superoxide to Oxygen and Hydrogen Peroxide in Carbon Nanoparticles. ACS Appl Mater Interf (2016) 8(24):15086–92. doi: 10.1021/acsami.6b03502 PMC4920082 27245481

[30] Vis B Hewitt RE Faria N Bastos C Chappell H Pele L. Non-Functionalized Ultrasmall Silica Nanoparticles Directly and Size-Selectively Activate T Cells. ACS Nano (2018) 12(11):10843–54. doi: 10.1021/acsnano.8b03363 30346692

[31] Thiramanas R Jiang S Simon J Landfester K Mailänder V. Silica Nanocapsules With Different Sizes and Physicochemical Properties as Suitable Nanocarriers for Uptake in T-Cells. Int J Nanomed (2020) 15:6069–84. doi: 10.2147/IJN.S246322 PMC7439283 32884263

[32] Thiramanas R Li M Jiang S Landfester K Mailänder V. Cellular Uptake of siRNA-Loaded Nanocarriers to Knockdown PD-L1: Strategies to Improve T-Cell Functions. Cells (2020) 9(9):1. doi: 10.3390/cells9092043 PMC7565787 32906726

[33] Tabujew I Willig M Leber N Freidel C Negwer I Koynov K. Overcoming the Barrier of CD8(+)T Cells: Two Types of Nano-Sized Carriers for siRNA Transport. Acta Biomater (2019) 100:338–51. doi: 10.1016/j.actbio.2019.10.006 31586726

[34] Tanaka H Miyama R Sakurai Y Tamagawa S Nakai Y Tange K. Improvement of mRNA Delivery Efficiency to a T Cell Line by Modulating PEG-Lipid Content and Phospholipid Components of Lipid Nanoparticles. Pharmaceutics (2021) 13(12):4. doi: 10.3390/pharmaceutics13122097 PMC8706876 34959378

[35] Tanaka H Takahashi T Konishi M Takata N Gomi M Shirane D. Self-Degradable Lipid-Like Materials Based on “Hydrolysis Accelerated by the Intra-Particle Enrichment of Reactant (HyPER)” for Messenger RNA Delivery. Adv Funct Mater (2020) 30(34):1910575. doi: 10.1002/adfm.201910575

[36] Dinauer N Balthasar S Weber C Kreuter J Langer K von Briesen H. Selective Targeting of Antibody-Conjugated Nanoparticles to Leukemic Cells and Primary T-Lymphocytes. Biomaterials (2005) 26(29):5898–906. doi: 10.1016/j.biomaterials.2005.02.038 15949555

[37] Kheirolomoom A Kare AJ Ingham ES Paulmurugan R Robinson ER Baikoghli M. *In Situ* T-Cell Transfection by Anti-CD3-Conjugated Lipid Nanoparticles Leads to T-Cell Activation, Migration, and Phenotypic Shift. Biomaterials (2022) 281:121339. doi: 10.1016/j.biomaterials.2021.121339 35078042 PMC8892572

[38] Ramana LN Sharma S Sethuraman S Ranga U Krishnan UM. Stealth Anti-CD4 Conjugated Immunoliposomes With Dual Antiretroviral Drugs–Modern Trojan Horses to Combat HIV. Eur J Pharm Biopharm (2015) 89:300–11. doi: 10.1016/j.ejpb.2014.11.021 25500283

[39] Ramishetti S Kedmi R Goldsmith M Leonard F Sprague AG Godin B. Systemic Gene Silencing in Primary T Lymphocytes Using Targeted Lipid Nanoparticles. ACS Nano (2015) 9(7):6706–16. doi: 10.1021/acsnano.5b02796 26042619

[40] Ermilova I Swenson J. DOPC Versus DOPE as a Helper Lipid for Gene-Therapies: Molecular Dynamics Simulations With DLin-MC3-DMA. Phys Chem Chem Phys (2020) 22(48):28256–68. doi: 10.1039/D0CP05111J 33295352

[41] Zheng Y Tang L Mabardi L Kumari S Irvine DJ. Enhancing Adoptive Cell Therapy of Cancer Through Targeted Delivery of Small-Molecule Immunomodulators to Internalizing or Noninternalizing Receptors. ACS Nano (2017) 11(3):3089–100. doi: 10.1021/acsnano.7b00078 PMC5647839 28231431

[42] Maier MA Jayaraman M Matsuda S Liu J Barros S Querbes W. Biodegradable Lipids Enabling Rapidly Eliminated Lipid Nanoparticles for Systemic Delivery of RNAi Therapeutics. Mol Ther (2013) 21(8):1570–8. doi: 10.1038/mt.2013.124 PMC3734658 23799535

[43] Tombácz I Laczkó D Shahnawaz H Muramatsu H Natesan A Yadegari A. Highly Efficient CD4+ T Cell Targeting and Genetic Recombination Using Engineered CD4+ Cell-Homing mRNA-LNPs. Mol Ther (2021) 29(11):3293–304. doi: 10.1016/j.ymthe.2021.06.004 PMC8571164 34091054

[44] Canakci M Singh K Munkhbat O Shanthalingam S Mitra A Gordon M. Targeting CD4(+) Cells With Anti-CD4 Conjugated Mertansine-Loaded Nanogels. Biomacromolecules (2020) 21(6):2473–81. doi: 10.1021/acs.biomac.0c00442 PMC7354862 32383874

[45] He F Wen N Xiao D Yan J Xiong H Cai S. Aptamer-Based Targeted Drug Delivery Systems: Current Potential and Challenges. Curr Med Chem (2020) 27(13):2189–219. doi: 10.2174/0929867325666181008142831 30295183

[46] Wang CW Chung WH Cheng YF Ying NW Peck K Chen YT. A New Nucleic Acid-Based Agent Inhibits Cytotoxic T Lymphocyte-Mediated Immune Disorders. J Allergy Clin Immunol (2013) 132(3):713–22.e11. doi: 10.1016/j.jaci.2013.04.036 23791505

[47] Luesakul U Puthong S Neamati N Muangsin N. pH-Responsive Selenium Nanoparticles Stabilized by Folate-Chitosan Delivering Doxorubicin for Overcoming Drug-Resistant Cancer Cells. Carbohydr Polym (2018) 181:841–50. doi: 10.1016/j.carbpol.2017.11.068 29254044

[48] Laskin BL Jiao J Baluarte HJ Amaral S Furth SL Akimova T. The Effects of Tacrolimus on T-Cell Proliferation Are Short-Lived: A Pilot Analysis of Immune Function Testing. Transplant Direct (2017) 3(8):e199. doi: 10.1097/TXD.0000000000000715 28795150 PMC5540637

[49] Mansouri A Abnous K Alibolandi M Taghdisi SM Ramezani M. Targeted Delivery of Tacrolimus to T Cells by pH-Responsive Aptamer-Chitosan- Poly(Lactic-Co-Glycolic Acid) Nanocomplex. J Cell Physiol (2019) 234(10):18262–71. doi: 10.1002/jcp.28458 30883749

[50] Glass JJ Yuen D Rae J Johnston AP Parton RG Kent SJ. Human Immune Cell Targeting of Protein Nanoparticles–Caveospheres. Nanoscale (2016) 8(15):8255–65. doi: 10.1039/C6NR00506C 27031090

[51] Frick SU Domogalla MP Baier G Wurm FR Mailänder V Landfester K. Interleukin-2 Functionalized Nanocapsules for T Cell-Based Immunotherapy. ACS Nano (2016) 10(10):9216–26. doi: 10.1021/acsnano.5b07973 27723299

[52] Gamrad L Rehbock C Westendorf AM Buer J Barcikowski S Hansen W. Efficient Nucleic Acid Delivery to Murine Regulatory T Cells by Gold Nanoparticle Conjugates. Sci Rep (2016) 6:28709. doi: 10.1038/srep28709 27381215 PMC4933883

[53] Li SY Liu Y Xu CF Shen S Sun R Du XJ. Restoring Anti-Tumor Functions of T Cells *via* Nanoparticle-Mediated Immune Checkpoint Modulation. J Control Release (2016) 231:17–28. doi: 10.1016/j.jconrel.2016.01.044 26829099

[54] Wu Y Gu W Li L Chen C Xu ZP. Enhancing PD-1 Gene Silence in T Lymphocytes by Comparing the Delivery Performance of Two Inorganic Nanoparticle Platforms. Nanomater (Basel) (2019) 9(2):2. doi: 10.3390/nano9020159 PMC6410115 30696033

[55] Barati M Mirzavi F Nikpoor AR Sankian M Namdar Ahmadabad H Soleimani A. Enhanced Antitumor Immune Response in Melanoma Tumor Model by Anti-PD-1 Small Interference RNA Encapsulated in Nanoliposomes. Cancer Gene Ther (2021). doi: 10.1038/s41417-021-00367-9 34341501

[56] Kosmides AK Sidhom JW Fraser A Bessell CA Schneck JP. Dual Targeting Nanoparticle Stimulates the Immune System To Inhibit Tumor Growth. ACS Nano (2017) 11(6):5417–29. doi: 10.1021/acsnano.6b08152 PMC8635119 28589725

[57] Ahmadzadeh M Johnson LA Heemskerk B Wunderlich JR Dudley ME White DE. Tumor Antigen-Specific CD8 T Cells Infiltrating the Tumor Express High Levels of PD-1 and are Functionally Impaired. Blood (2009) 114(8):1537–44. doi: 10.1182/blood-2008-12-195792 PMC2927090 19423728

[58] Chester C Sanmamed MF Wang J Melero I. Immunotherapy Targeting 4-1BB: Mechanistic Rationale, Clinical Results, and Future Strategies. Blood (2018) 131(1):49–57. doi: 10.1182/blood-2017-06-741041 29118009

[59] Yang YS Moynihan KD Bekdemir A Dichwalkar TM Noh MM Watson N. Targeting Small Molecule Drugs to T Cells With Antibody-Directed Cell-Penetrating Gold Nanoparticles. Biomater Sci (2018) 7(1):113–24. doi: 10.1039/C8BM01208C PMC6310171 30444251

[60] Francis DM Manspeaker MP Archer PA Sestito LF Heiler AJ Schudel A. Drug-Eluting Immune Checkpoint Blockade Antibody-Nanoparticle Conjugate Enhances Locoregional and Systemic Combination Cancer Immunotherapy Through T Lymphocyte Targeting. Biomaterials (2021) 279:121184. doi: 10.1016/j.biomaterials.2021.121184 34678650 PMC8639654

[61] Cluxton D Petrasca A Moran B Fletcher JM. Differential Regulation of Human Treg and Th17 Cells by Fatty Acid Synthesis and Glycolysis. Front Immunol (2019) 10:115. doi: 10.3389/fimmu.2019.00115 30778354 PMC6369198

[62] Muroski ME Miska J Chang AL Zhang P Rashidi A Moore H. Fatty Acid Uptake in T Cell Subsets Using a Quantum Dot Fatty Acid Conjugate. Sci Rep (2017) 7(1):5790. doi: 10.1038/s41598-017-05556-x 28724939 PMC5517517

[63] Kim D Wu Y Li Q Oh YK. Nanoparticle-Mediated Lipid Metabolic Reprogramming of T Cells in Tumor Microenvironments for Immunometabolic Therapy. Nanomicro Lett (2021) 13(1):31. doi: 10.1007/s40820-020-00555-6 34138236 PMC8006499

[64] Bam M Chintala S Fetcko K Williamsen BC Siraj S Liu S. Genome Wide DNA Methylation Landscape Reveals Glioblastoma's Influence on Epigenetic Changes in Tumor Infiltrating CD4+ T Cells. Oncotarget (2021) 12(10):967–81. doi: 10.18632/oncotarget.27955 PMC8121608 34012510

[65] Hu N Li W Hong Y Zeng Z Zhang J Wu X. A PD1 Targeted Nano-Delivery System Based on Epigenetic Alterations of T Cell Responses in the Treatment of Gastric Cancer. Mol Ther Oncolytics (2022) 24:148–59. doi: 10.1016/j.omto.2021.12.006 PMC8724952 35024441

[66] Wang JX Choi SYC Niu X Kang N Xue H Killam J. Lactic Acid and an Acidic Tumor Microenvironment Suppress Anticancer Immunity. Int J Mol Sci (2020) 21(21):2. doi: 10.3390/ijms21218363 PMC7664620 33171818

[67] Zhang YX Zhao YY Shen J Sun X Liu Y Liu H. Nanoenabled Modulation of Acidic Tumor Microenvironment Reverses Anergy of Infiltrating T Cells and Potentiates Anti-PD-1 Therapy. Nano Lett (2019) 19(5):2774–83. doi: 10.1021/acs.nanolett.8b04296 30943039

[68] Hei Y Teng B Zeng Z Zhang S Li Q Pan J. Multifunctional Immunoliposomes Combining Catalase and PD-L1 Antibodies Overcome Tumor Hypoxia and Enhance Immunotherapeutic Effects Against Melanoma. Int J Nanomed (2020) 15:1677–91. doi: 10.2147/IJN.S225807 PMC7082626 32214807

[69] Li T Kang G Wang T Huang H. Tumor Angiogenesis and Anti-Angiogenic Gene Therapy for Cancer. Oncol Lett (2018) 16(1):687–702. doi: 10.3892/ol.2018.8733 29963134 PMC6019900

[70] Hei Y Chen Y Li Q Mei Z Pan J Zhang S. Multifunctional Immunoliposomes Enhance the Immunotherapeutic Effects of PD-L1 Antibodies Against Melanoma by Reprogramming Immunosuppressive Tumor Microenvironment. Small (2022) 18(9):e2105118. doi: 10.1002/smll.202105118 34915595

[71] Russ A Hua AB Montfort WR Rahman B Riaz IB Khalid MU. Blocking "Don't Eat Me" Signal of CD47-Sirpα in Hematological Malignancies, an in-Depth Review. Blood Rev (2018) 32(6):480–9. doi: 10.1016/j.blre.2018.04.005 PMC6186508 29709247

[72] Labanieh L Majzner RG Mackall CL. Programming CAR-T Cells to Kill Cancer. Nat BioMed Eng (2018) 2(6):377–91. doi: 10.1038/s41551-018-0235-9 31011197

[73] Dana H Chalbatani GM Jalali SA Mirzaei HR Grupp SA Suarez ER. CAR-T Cells: Early Successes in Blood Cancer and Challenges in Solid Tumors. Acta Pharm Sin B (2021) 11(5):1129–47. doi: 10.1016/j.apsb.2020.10.020 PMC8144892 34094824

[74] Lukjanov V Koutná I Šimara P. CAR T-Cell Production Using Nonviral Approaches. J Immunol Res (2021) 2021:6644685. doi: 10.1155/2021/6644685 33855089 PMC8019376

[75] Billingsley MM Singh N Ravikumar P Zhang R June CH Mitchell MJ. Ionizable Lipid Nanoparticle-Mediated mRNA Delivery for Human CAR T Cell Engineering. Nano Lett (2020) 20(3):1578–89. doi: 10.1021/acs.nanolett.9b04246 PMC7313236 31951421

[76] Billingsley MM Hamilton AG Mai D Patel SK Swingle KL Sheppard NC. Orthogonal Design of Experiments for Optimization of Lipid Nanoparticles for mRNA Engineering of CAR T Cells. Nano Lett (2022) 22(1):533–42. doi: 10.1021/acs.nanolett.1c02503 PMC9335860 34669421

[77] Zhao X Chen J Qiu M Li Y Glass Z Xu Q. Imidazole-Based Synthetic Lipidoids for In Vivo mRNA Delivery Into Primary T Lymphocytes. Angew Chem Int Ed Engl (2020) 59(45):20083–9. doi: 10.1002/anie.202008082 32662132

[78] Ye Z Chen J Zhao X Li Y Harmon J Huang C. *In Vitro* Engineering Chimeric Antigen Receptor Macrophages and T Cells by Lipid Nanoparticle-Mediated mRNA Delivery. ACS Biomater Sci Eng (2022) 8(2):722–33. doi: 10.1021/acsbiomaterials.1c01532 35104103

[79] Yu Q Zhang M Chen Y Chen X Shi S Sun K. Self-Assembled Nanoparticles Prepared From Low-Molecular-Weight PEI and Low-Generation PAMAM for EGFRvIII-Chimeric Antigen Receptor Gene Loading and T-Cell Transient Modification. Int J Nanomed (2020) 15:483–95. doi: 10.2147/IJN.S229858 PMC6986680 32158206

[80] Kiru L Zlitni A Tousley AM Dalton GN Wu W Lafortune F. *In Vivo* Imaging of Nanoparticle-Labeled CAR T Cells. Proc Natl Acad Sci USA (2022) 119(6):1. doi: 10.1073/pnas.2102363119 PMC8832996 35101971

[81] Zhang W Gaikwad H Groman EV Purev E Simberg D Wang G. Highly Aminated Iron Oxide Nanoworms for Simultaneous Manufacturing and Labeling of Chimeric Antigen Receptor T Cells. J Magn Magn Mater (2022) 541:1. doi: 10.1016/j.jmmm.2021.168480 PMC8553019 34720339

[82] Luo Y Chen Z Sun M Li B Pan F Ma A. IL-12 Nanochaperone-Engineered CAR T Cell for Robust Tumor-Immunotherapy. Biomaterials (2022) 281:121341. doi: 10.1016/j.biomaterials.2021.121341 34995901

[83] Larkin J Chiarion-Sileni V Gonzalez R Grob JJ Cowey CL Lao CD. Combined Nivolumab and Ipilimumab or Monotherapy in Untreated Melanoma. N Engl J Med (2015) 373(1):23–34. doi: 10.1056/NEJMoa1504030 26027431 PMC5698905

[84] Antonia SJ López-Martin JA Bendell J Ott PA Taylor M Eder JP. Nivolumab Alone and Nivolumab Plus Ipilimumab in Recurrent Small-Cell Lung Cancer (CheckMate 032): A Multicentre, Open-Label, Phase 1/2 Trial. Lancet Oncol (2016) 17(7):883–95. doi: 10.1016/S1470-2045(16)30098-5 27269741

[85] Kaufman HL Russell J Hamid O Bhatia S Terheyden P D'Angelo SP. Avelumab in Patients With Chemotherapy-Refractory Metastatic Merkel Cell Carcinoma: A Multicentre, Single-Group, Open-Label, Phase 2 Trial. Lancet Oncol (2016) 17(10):1374–85. doi: 10.1016/S1470-2045(16)30364-3 PMC5587154 27592805

[86] Overman MJ McDermott R Leach JL Lonardi S Lenz HJ Morse MA. Nivolumab in Patients With Metastatic DNA Mismatch Repair-Deficient or Microsatellite Instability-High Colorectal Cancer (CheckMate 142): An Open-Label, Multicentre, Phase 2 Study. Lancet Oncol (2017) 18(9):1182–91. doi: 10.1016/S1470-2045(17)30422-9 PMC6207072 28734759

[87] Ferris RL Blumenschein G Fayette J Guigay J Colevas AD Licitra L. Nivolumab for Recurrent Squamous-Cell Carcinoma of the Head and Neck. New Engl J Med (2016) 375(19):1856–67. doi: 10.1056/NEJMoa1602252 PMC5564292 27718784

[88] Sharma P Retz M Siefker-Radtke A Baron A Necchi A Bedke J. Nivolumab in Metastatic Urothelial Carcinoma After Platinum Therapy (CheckMate 275): A Multicentre, Single-Arm, Phase 2 Trial. Lancet Oncol (2017) 18(3):312–22. doi: 10.1016/S1470-2045(17)30065-7 28131785

[89] Motzer RJ Rini BI McDermott DF Redman BG Kuzel TM Harrison MR. Nivolumab for Metastatic Renal Cell Carcinoma: Results of a Randomized Phase II Trial. J Clin Oncol (2015) 33(13):1430–7. doi: 10.1200/JCO.2014.59.0703 PMC4806782 25452452

[90] Majzner RG Mackall CL. Clinical Lessons Learned From the First Leg of the CAR T Cell Journey. Nat Med (2019) 25(9):1341–55. doi: 10.1038/s41591-019-0564-6 31501612

[91] Neelapu SS Locke FL Bartlett NL Lekakis LJ Miklos DB Jacobson CA. Axicabtagene Ciloleucel CAR T-Cell Therapy in Refractory Large B-Cell Lymphoma. N Engl J Med (2017) 377(26):2531–44. doi: 10.1056/NEJMoa1707447 PMC5882485 29226797

[92] Abramson JS Palomba ML Gordon LI Lunning MA Wang M Arnason J. Lisocabtagene Maraleucel for Patients With Relapsed or Refractory Large B-Cell Lymphomas (TRANSCEND NHL 001): A Multicentre Seamless Design Study. Lancet (2020) 396(10254):839–52. doi: 10.1016/S0140-6736(20)31366-0 32888407

[93] Schuster SJ Bishop MR Tam CS Waller EK Borchmann P McGuirk JP. Tisagenlecleucel in Adult Relapsed or Refractory Diffuse Large B-Cell Lymphoma. N Engl J Med (2019) 380(1):45–56. doi: 10.1056/NEJMoa1804980 30501490

[94] Jenkins RW Barbie DA Flaherty KT. Mechanisms of Resistance to Immune Checkpoint Inhibitors. Br J Cancer (2018) 118(1):9–16. doi: 10.1038/bjc.2017.434 29319049 PMC5765236

[95] Fesnak AD June CH Levine BL. Engineered T Cells: The Promise and Challenges of Cancer Immunotherapy. Nat Rev Cancer (2016) 16(9):566–81. doi: 10.1038/nrc.2016.97 PMC5543811 27550819

[96] Fridman WH Pages F Sautes-Fridman C Galon J. The Immune Contexture in Human Tumours: Impact on Clinical Outcome. Nat Rev Cancer (2012) 12(4):298–306. doi: 10.1038/nrc3245 22419253

[97] Debele TA Yeh CF Su WP. Cancer Immunotherapy and Application of Nanoparticles in Cancers Immunotherapy as the Delivery of Immunotherapeutic Agents and as the Immunomodulators. Cancers (Basel) (2020) 12(12):3773. doi: 10.3390/cancers12123773 PMC7765190 33333816

[98] Shi Y. Clinical Translation of Nanomedicine and Biomaterials for Cancer Immunotherapy: Progress and Perspectives. Adv Ther (2020) 3(9):1900215. doi: 10.1002/adtp.201900215

[99] Gargett T Yu W Dotti G Yvon ES Christo SN Hayball JD. GD2-Specific CAR T Cells Undergo Potent Activation and Deletion Following Antigen Encounter But can be Protected From Activation-Induced Cell Death by PD-1 Blockade. Mol Ther (2016) 24(6):1135–49. doi: 10.1038/mt.2016.63 PMC4923328 27019998

[100] Patel MR Bauer TM Jimeno A Wang D LoRusso P Do KT. A Phase I Study of mRNA-2752, a Lipid Nanoparticle Encapsulating mRNAs Encoding Human OX40L, IL-23, and IL-36γ, for Intratumoral (iTu) Injection Alone and in Combination With Durvalumab. J Clin Oncol (2020) 38(15_suppl):3092. doi: 10.1200/JCO.2020.38.15_suppl.3092

[101] Bechter O Utikal J Baurain J-F Massard C Sahin U Derhovanessian E. 391 A First-in-Human Study of Intratumoral SAR441000, an mRNA Mixture Encoding IL-12sc, Interferon Alpha2b, GM-CSF and IL-15sushi as Monotherapy and in Combination With Cemiplimab in Advanced Solid Tumors. J ImmunoTher Cancer (2020) 8(Suppl 3):A237–A8. doi: 10.1136/jitc-2020-SITC2020.0391

[102] Butler MO Friedlander P Milstein MI Mooney MM Metzler G Murray AP. Establishment of Antitumor Memory in Humans Using *In Vitro*-Educated CD8+ T Cells. Sci Transl Med (2011) 3(80):80ra34. doi: 10.1126/scitranslmed.3002207 PMC3861895 21525398

[103] Shen L Tenzer S Storck W Hobernik D Raker VK Fischer K. Protein corona-mediated targeting of nanocarriers to B cells allows redirection of allergic immune responses. J Allergy Clin Immunol (2018) 142(5):1558–70. doi: 10.1016/j.jaci.2017.08.049 29382591

[104] Kang C Syed YY. Atezolizumab (in Combination With Nab-Paclitaxel): A Review in Advanced Triple-Negative Breast Cancer. Drugs (2020) 80(6):601–7. doi: 10.1007/s40265-020-01295-y 32248356

[105] West H McCleod M Hussein M Morabito A Rittmeyer A Conter HJ. Atezolizumab in Combination With Carboplatin Plus Nab-Paclitaxel Chemotherapy Compared With Chemotherapy Alone as First-Line Treatment for Metastatic non-Squamous non-Small-Cell Lung Cancer (IMpower130): A Multicentre, Randomised, Open-Label, Phase 3 Trial. Lancet Oncol (2019) 20(7):924–37. doi: 10.1016/S1470-2045(19)30167-6 31122901

[106] Pujade-Lauraine E Fujiwara K Dychter SS Devgan G Monk BJ. Avelumab (Anti-PD-L1) in Platinum-Resistant/Refractory Ovarian Cancer: JAVELIN Ovarian 200 Phase III Study Design. Future Oncol (2018) 14(21):2103–13. doi: 10.2217/fon-2018-0070 29584456

[107] Wang-Gillam A Li CP Bodoky G Dean A Shan YS Jameson G. Nanoliposomal Irinotecan With Fluorouracil and Folinic Acid in Metastatic Pancreatic Cancer After Previous Gemcitabine-Based Therapy (NAPOLI-1): A Global, Randomised, Open-Label, Phase 3 Trial. Lancet (2016) 387(10018):545–57. doi: 10.1016/s0140-6736(15)00986-1 26615328

[108] Sahin U Oehm P Derhovanessian E Jabulowsky RA Vormehr M Gold M. An RNA Vaccine Drives Immunity in Checkpoint-Inhibitor-Treated Melanoma. Nature (2020) 585(7823):107–12. doi: 10.1038/s41586-020-2537-9 32728218

[109] Kranz LM Diken M Haas H Kreiter S Loquai C Reuter KC. Systemic RNA Delivery to Dendritic Cells Exploits Antiviral Defence for Cancer Immunotherapy. Nature (2016) 534(7607):396–401. doi: 10.1038/nature18300 27281205

[110] Burris Iii HA Patel MR Cho DC Clarke JM Gutierrez M Zaks TZ. A Phase 1, Open-Label, Multicenter Study to Assess the Safety, Tolerability, and Immunogenicity of mRNA-4157 Alone in Subjects With Resected Solid Tumors and in Combination With Pembrolizumab in Subjects With Unresectable Solid Tumors (Keynote-603). J Global Oncol (2019) 5(suppl):93. doi: 10.1200/JGO.2019.5.suppl.93

[111] Pfannenstiel LW Lam SS Emens LA Jaffee EM Armstrong TD. Paclitaxel Enhances Early Dendritic Cell Maturation and Function Through TLR4 Signaling in Mice. Cell Immunol (2010) 263(1):79–87. doi: 10.1016/j.cellimm.2010.03.001 20346445 PMC2862830

[112] Graziani SR Vital CG Morikawa AT Van Eyll BM Fernandes Junior HJ Kalil Filho R. Phase II Study of Paclitaxel Associated With Lipid Core Nanoparticles (LDE) as Third-Line Treatment of Patients With Epithelial Ovarian Carcinoma. Med Oncol (2017) 34(9):151. doi: 10.1007/s12032-017-1009-z 28756613

[113] Yamaguchi K Iwasa S Hirao M Oshima T Harada K Sato Y. Phase 1 Study of the Liposomal Formulation of Eribulin (E7389-LF): Results From the Advanced Gastric Cancer Expansion Cohort. J Clin Oncol (2021) 39(15):4025. doi: 10.1200/JCO.2021.39.15_suppl.4025 PMC10102841 36730323

[114] Linch M Papai Z Takacs I Imedio ER Kühnle M-C Derhovanessian E. 421 A First-in-Human (FIH) Phase I/IIa Clinical Trial Assessing a Ribonucleic Acid Lipoplex (RNA-LPX) Encoding Shared Tumor Antigens for Immunotherapy of Prostate Cancer; Preliminary Analysis of PRO-MERIT. J ImmunoTher Cancer (2021) 9(Suppl 2):A451–A. doi: 10.1136/jitc-2021-SITC2021.421

[115] Grunwitz C Salomon N Vascotto F Selmi A Bukur T Diken M. HPV16 RNA-LPX Vaccine Mediates Complete Regression of Aggressively Growing HPV-Positive Mouse Tumors and Establishes Protective T Cell Memory. Oncoimmunology (2019) 8(9):e1629259. doi: 10.1080/2162402X.2019.1629259 31428528 PMC6685602

[116] Schmidt M Bolte S Frenzel K Heesen L Derhovanessian E Bukur V. Abstract OT2-06-01: Highly Innovative Personalized RNA-Immunotherapy for Patients With Triple Negative Breast Cancer. Cancer Res (2019) 79(4_Supplement):OT2-06-1-OT2–1. doi: 10.1158/1538-7445.SABCS18-OT2-06-01

[117] Braiteh F LoRusso P Balmanoukian A Klempner S Camidge DR Hellmann M. Abstract CT169: A Phase Ia Study to Evaluate RO7198457, an Individualized Neoantigen Specific Immunotherapy (Inest), in Patients With Locally Advanced or Metastatic Solid Tumors. Cancer Res (2020) 80(16_Supplement):CT169–CT. doi: 10.1158/1538-7445.AM2020-CT169

[118] Haydon A Alamgeer M Brungs D Collichio F Khushalani N Colevas D. 547 CERPASS: A Randomized, Controlled, Open-Label, Phase 2 Study of Cemiplimab ± RP1 in Patients With Advanced Cutaneous Squamous Cell Carcinoma. J ImmunoTher Cancer (2021) 9(Suppl 2):A577–A. doi: 10.1136/jitc-2021-SITC2021.547

[119] Shen C Frakes J Weiss J Caudell JJ Hackman TG Akulian JA. Phase I Study of NBTXR3 Activated by Radiotherapy in Patients With Advanced Cancers Treated With an Anti-PD-1 Therapy. J Clin Oncol (2020) 38(15):TPS3173–TPS. doi: 10.1200/JCO.2020.38.15_suppl.TPS3173

[120] Bonvalot S Rutkowski PL Thariat J Carrere S Ducassou A Sunyach MP. NBTXR3, a First-in-Class Radioenhancer Hafnium Oxide Nanoparticle, Plus Radiotherapy Versus Radiotherapy Alone in Patients With Locally Advanced Soft-Tissue Sarcoma (Act.In.Sarc): A Multicentre, Phase 2-3, Randomised, Controlled Trial. Lancet Oncol (2019) 20(8):1148–59. doi: 10.1016/S1470-2045(19)30326-2 31296491

[121] Shan X Gong X Li J Wen J Li Y Zhang Z. Current Approaches of Nanomedicines in the Market and Various Stage of Clinical Translation. Acta Pharm Sin B (2022). doi: 10.1016/j.apsb.2022.02.025 PMC9293719 35865096

[122] Demaria S Volm MD Shapiro RL Yee HT Oratz R Formenti SC. Development of Tumor-Infiltrating Lymphocytes in Breast Cancer After Neoadjuvant Paclitaxel Chemotherapy1. Clin Cancer Res (2001) 7(10):3025–30. 11595690

[123] Vicari AP Luu R Zhang N Patel S Makinen SR Hanson DC. Paclitaxel Reduces Regulatory T Cell Numbers and Inhibitory Function and Enhances the Anti-Tumor Effects of the TLR9 Agonist PF-3512676 in the Mouse. Cancer Immunol Immunother (2009) 58(4):615–28. doi: 10.1007/s00262-008-0586-2 PMC11030133 18802696

[124] Ran S. The Role of TLR4 in Chemotherapy-Driven Metastasis. Cancer Res (2015) 75(12):2405–10. doi: 10.1158/0008-5472.CAN-14-3525 PMC4470764 25998620

[125] Gradishar WJ Tjulandin S Davidson N Shaw H Desai N Bhar P. Phase III Trial of Nanoparticle Albumin-Bound Paclitaxel Compared With Polyethylated Castor Oil-Based Paclitaxel in Women With Breast Cancer. J Clin Oncol (2005) 23(31):7794–803. doi: 10.1200/JCO.2005.04.937 16172456

[126] Schmid P Adams S Rugo HS Schneeweiss A Barrios CH Iwata H. Atezolizumab and Nab-Paclitaxel in Advanced Triple-Negative Breast Cancer. N Engl J Med (2018) 379(22):2108–21. doi: 10.1056/NEJMoa1809615 30345906

[127] Dirix LY Takacs I Jerusalem G Nikolinakos P Arkenau HT Forero-Torres A. Avelumab, an Anti-PD-L1 Antibody, in Patients With Locally Advanced or Metastatic Breast Cancer: A Phase 1b JAVELIN Solid Tumor Study. Breast Cancer Res Treat (2018) 167(3):671–86. doi: 10.1007/s10549-017-4537-5 PMC5807460 29063313

[128] Emens LA Cruz C Eder JP Braiteh F Chung C Tolaney SM. Long-Term Clinical Outcomes and Biomarker Analyses of Atezolizumab Therapy for Patients With Metastatic Triple-Negative Breast Cancer: A Phase 1 Study. JAMA Oncol (2019) 5(1):74–82. doi: 10.1001/jamaoncol.2018.4224 30242306 PMC6439773

[129] Paz-Ares L Luft A Vicente D Tafreshi A Gumus M Mazieres J. Pembrolizumab Plus Chemotherapy for Squamous Non-Small-Cell Lung Cancer. N Engl J Med (2018) 379(21):2040–51. doi: 10.1056/NEJMoa1810865 30280635

[130] Aigner J Marme F Smetanay K Schuetz F Jaeger D Schneeweiss A. Nab-Paclitaxel Monotherapy as a Treatment of Patients With Metastatic Breast Cancer in Routine Clinical Practice. Anticancer Res (2013) 33(8):3407–13. 23898112

[131] Zhang Z Zhang Y Song S Yin L Sun D Gu J. Recent Advances in the Bioanalytical Methods of Polyethylene Glycols and PEGylated Pharmaceuticals. J Separation Sci (2020) 43(9-10):1978–97. doi: 10.1002/jssc.201901340 32077620

[132] Alizadeh D Trad M Hanke NT Larmonier CB Janikashvili N Bonnotte B. Doxorubicin Eliminates Myeloid-Derived Suppressor Cells and Enhances the Efficacy of Adoptive T-Cell Transfer in Breast Cancer. Cancer Res (2014) 74(1):104–18. doi: 10.1158/0008-5472.CAN-13-1545 PMC3896092 24197130

[133] Ramakrishnan R Assudani D Nagaraj S Hunter T Cho HI Antonia S. Chemotherapy Enhances Tumor Cell Susceptibility to CTL-Mediated Killing During Cancer Immunotherapy in Mice. J Clin Invest (2010) 120(4):1111–24. doi: 10.1172/JCI40269 PMC2846048 20234093

[134] Obeid M Tesniere A Ghiringhelli F Fimia GM Apetoh L Perfettini JL. Calreticulin Exposure Dictates the Immunogenicity of Cancer Cell Death. Nat Med (2007) 13(1):54–61. doi: 10.1038/nm1523 17187072

[135] Rios-Doria J Durham N Wetzel L Rothstein R Chesebrough J Holoweckyj N. Doxil Synergizes With Cancer Immunotherapies to Enhance Antitumor Responses in Syngeneic Mouse Models. Neoplasia (2015) 17(8):661–70. doi: 10.1016/j.neo.2015.08.004 PMC4674486 26408258

[136] Shi Y Lammers T. Combining Nanomedicine and Immunotherapy. Acc Chem Res (2019) 52(6):1543–54. doi: 10.1021/acs.accounts.9b00148 PMC7115879 31120725

[137] Marina NM Cochrane D Harney E Zomorodi K Blaney S Winick N. Dose Escalation and Pharmacokinetics of Pegylated Liposomal Doxorubicin (Doxil) in Children With Solid Tumors: A Pediatric Oncology Group Study1. Clin Cancer Res (2002) 8(2):413–8. 11839657

[138] O'Brien ME Wigler N Inbar M Rosso R Grischke E Santoro A. Reduced Cardiotoxicity and Comparable Efficacy in a Phase III Trial of Pegylated Liposomal Doxorubicin HCl (CAELYX/Doxil) Versus Conventional Doxorubicin for First-Line Treatment of Metastatic Breast Cancer. Ann Oncol (2004) 15(3):440–9. doi: 10.1093/annonc/mdh097 14998846

[139] Lee EK Xiong N Cheng SC Barry WT Penson RT Konstantinopoulos PA. Combined Pembrolizumab and Pegylated Liposomal Doxorubicin in Platinum Resistant Ovarian Cancer: A Phase 2 Clinical Trial. Gynecol Oncol (2020) 159(1):72–8. doi: 10.1016/j.ygyno.2020.07.028 32771276

[140] Kyte JA Rossevold A Falk RS Naume B. ALICE: A Randomized Placebo-Controlled Phase II Study Evaluating Atezolizumab Combined With Immunogenic Chemotherapy in Patients With Metastatic Triple-Negative Breast Cancer. J Transl Med (2020) 18(1):252. doi: 10.1186/s12967-020-02424-7 32576225 PMC7310523

[141] Kyte JA Andresen NK Russnes HG Fretland SO Falk RS Lingjaerde OC. ICON: A Randomized Phase IIb Study Evaluating Immunogenic Chemotherapy Combined With Ipilimumab and Nivolumab in Patients With Metastatic Hormone Receptor Positive Breast Cancer. J Transl Med (2020) 18(1):269. doi: 10.1186/s12967-020-02421-w 32620163 PMC7333428

[142] Little RF Aleman K Kumar P Wyvill KM Pluda JM Read-Connole E. Phase 2 Study of Pegylated Liposomal Doxorubicin in Combination With Interleukin-12 for AIDS-Related Kaposi Sarcoma. Blood (2007) 110(13):4165–71. doi: 10.1182/blood-2007-06-097568 PMC2234790 17846226

[143] Simpkins F Flores AM Chu C Lucci JA Berek JS Coukos G. Dose Escalation Trial to Assess the Safety and Biological Activity of Recombinant Human Interleukin-18 (SB-485232) in Combination With Pegylated Liposomal Doxorubicin in Platinum-Resistant Recurrent Ovarian Cancer. J Clin Oncol (2012) 30(15):5065. doi: 10.1200/jco.2012.30.15_suppl.5065

[144] Pardi N Hogan MJ Porter FW Weissman D. mRNA Vaccines - a New Era in Vaccinology. Nat Rev Drug Discovery (2018) 17(4):261–79. doi: 10.1038/nrd.2017.243 PMC5906799 29326426

[145] Melero I Gaudernack G Gerritsen W Huber C Parmiani G Scholl S. Therapeutic Vaccines for Cancer: An Overview of Clinical Trials. Nat Rev Clin Oncol (2014) 11(9):509–24. doi: 10.1038/nrclinonc.2014.111 25001465

[146] Kowalski PS Rudra A Miao L Anderson DG. Delivering the Messenger: Advances in Technologies for Therapeutic mRNA Delivery. Mol Ther (2019) 27(4):710–28. doi: 10.1016/j.ymthe.2019.02.012 PMC6453548 30846391

[147] Kruit W Suciu S Dreno B Mortier L Robert C Chiarion-Sileni V. Selection of Immunostimulant AS15 for Active Immunization With MAGE-A3 Protein: Results of a Randomized Phase II Study of the European Organisation for Research and Treatment of Cancer Melanoma Group in Metastatic Melanoma. J Clin Oncol (2013) 31(19):2413–20. doi: 10.1200/JCO.2012.43.7111 23715572

[148] Vansteenkiste J Cho B Vanakesa T De Pas T Zielinski M Kim M. MAGRIT, a Double-Blind, Randomized, Placebo-Controlled Phase III Study to Assess the Efficacy of the recMAGE-A3+ AS15 Cancer Immunotherapeutic as Adjuvant Therapy in Patients With Resected MAGE-A3-Positive non-Small Cell Lung Cancer (NSCLC). Ann Oncol (2014) 25:iv409. doi: 10.1093/annonc/mdu347.1 27132212

[149] Berinstein NL Karkada M Morse MA Nemunaitis JJ Chatta G Kaufman H. First-In-Man Application of a Novel Therapeutic Cancer Vaccine Formulation With the Capacity to Induce Multi-Functional T Cell Responses in Ovarian, Breast and Prostate Cancer Patients. J Transl Med (2012) 10:156. doi: 10.1186/1479-5876-10-156 22862954 PMC3479010

[150] Berinstein NL Karkada M Oza AM Odunsi K Villella JA Nemunaitis JJ. Survivin-Targeted Immunotherapy Drives Robust Polyfunctional T Cell Generation and Differentiation in Advanced Ovarian Cancer Patients. OncoImmunology (2015) 4(8):e1026529. doi: 10.1080/2162402X.2015.1026529 26405584 PMC4570133

[151] Berinstein NL Bence-Bruckler I Laneuville P Stewart DA Forward NA Smyth L. Combination of DPX-Survivac, Low Dose Cyclophosphamide, and Pembrolizumab in Recurrent/Refractory DLBCL: The Spirel Study. Blood (2019) 134(Supplement_1):3236. doi: 10.1182/blood-2019-125963

[152] Senzer NN Kaufman HL Amatruda T Nemunaitis M Reid T Daniels G. Phase II Clinical Trial of a Granulocyte-Macrophage Colony-Stimulating Factor-Encoding, Second-Generation Oncolytic Herpesvirus in Patients With Unresectable Metastatic Melanoma. J Clin Oncol (2009) 27(34):5763–71. doi: 10.1200/JCO.2009.24.3675 19884534

[153] Ferrucci PF Pala L Conforti F Cocorocchio E. Talimogene Laherparepvec (T-VEC): An Intralesional Cancer Immunotherapy for Advanced Melanoma. Cancers (Basel) (2021) 13(6):1383. doi: 10.3390/cancers13061383 33803762 PMC8003308

[154] Andtbacka RH Ross MI Agarwala SS Taylor MH Vetto JT Neves RI. Final Results of a Phase II Multicenter Trial of HF10, a Replication-Competent HSV-1 Oncolytic Virus, and Ipilimumab Combination Treatment in Patients With Stage IIIB-IV Unresectable or Metastatic Melanoma. J Clin Oncol (2017) 35(15_suppl):9510. doi: 10.1200/JCO.2017.35.15_suppl.9510

[155] Ribas A Dummer R Puzanov I VanderWalde A Andtbacka RHI Michielin O. Oncolytic Virotherapy Promotes Intratumoral T Cell Infiltration and Improves Anti-PD-1 Immunotherapy. Cell (2017) 170(6):1109–19.e10. doi: 10.1016/j.cell.2017.08.027 28886381 PMC8034392

[156] Malvehy J Samoylenko I Schadendorf D Gutzmer R Grob JJ Sacco JJ. Talimogene Laherparepvec Upregulates Immune-Cell Populations in non-Injected Lesions: Findings From a Phase II, Multicenter, Open-Label Study in Patients With Stage IIIB-IVM1c Melanoma. J Immunother Cancer (2021) 9(3):1. doi: 10.1136/jitc-2020-001621 PMC8011715 33785610

[157] Chinnasamy D Tran E Yu Z Morgan RA Restifo NP Rosenberg SA. Simultaneous Targeting of Tumor Antigens and the Tumor Vasculature Using T Lymphocyte Transfer Synergize to Induce Regression of Established Tumors in Mice. Cancer Res (2013) 73(11):3371–80. doi: 10.1158/0008-5472.CAN-12-3913 PMC3686092 23633494

[158] Nazha B Inal C Owonikoko TK. Disialoganglioside GD2 Expression in Solid Tumors and Role as a Target for Cancer Therapy. Front Oncol (2020) 10:1000. doi: 10.3389/fonc.2020.01000 32733795 PMC7358363

[159] Tanoue K Rosewell Shaw A Watanabe N Porter C Rana B Gottschalk S. Armed Oncolytic Adenovirus–Expressing PD-L1 Mini-Body Enhances Antitumor Effects of Chimeric Antigen Receptor T Cells in Solid Tumors. Cancer Res (2017) 77(8):2040–51. doi: 10.1158/0008-5472.CAN-16-1577 PMC5392365 28235763

[160] Chapuis AG Roberts IM Thompson JA Margolin KA Bhatia S Lee SM. T-Cell Therapy Using Interleukin-21-Primed Cytotoxic T-Cell Lymphocytes Combined With Cytotoxic T-Cell Lymphocyte Antigen-4 Blockade Results in Long-Term Cell Persistence and Durable Tumor Regression. J Clin Oncol (2016) 34(31):3787–95. doi: 10.1200/JCO.2015.65.5142 PMC5477923 27269940

[161] Soltantoyeh T Akbari B Karimi A Mahmoodi Chalbatani G Ghahri-Saremi N Hadjati J. Chimeric Antigen Receptor (CAR) T Cell Therapy for Metastatic Melanoma: Challenges and Road Ahead. Cells (2021) 10(6):1450. doi: 10.3390/cells10061450 34207884 PMC8230324

[162] Butts C Murray N Maksymiuk A Goss G Marshall E Soulières D. Randomized Phase IIB Trial of BLP25 Liposome Vaccine in Stage IIIB and IV non-Small-Cell Lung Cancer. J Clin Oncol (2005) 23(27):6674–81. doi: 10.1200/JCO.2005.13.011 16170175

[163] ten Brinke A Karsten ML Dieker MC Zwaginga JJ van Ham SM. The Clinical Grade Maturation Cocktail Monophosphoryl Lipid A Plus Ifnγ Generates Monocyte-Derived Dendritic Cells With the Capacity to Migrate and Induce Th1 Polarization. Vaccine (2007) 25(41):7145–52. doi: 10.1016/j.vaccine.2007.07.031 17719152

[164] Butts C Socinski MA Mitchell PL Thatcher N Havel L Krzakowski M. Tecemotide (L-BLP25) Versus Placebo After Chemoradiotherapy for Stage III non-Small-Cell Lung Cancer (START): A Randomised, Double-Blind, Phase 3 Trial. Lancet Oncol (2014) 15(1):59–68. doi: 10.1016/S1470-2045(13)70510-2 24331154

[165] Schimanski CC Kasper S Hegewisch-Becker S Schröder J Overkamp F Kullmann F. Adjuvant MUC Vaccination With Tecemotide After Resection of Colorectal Liver Metastases: A Randomized, Double-Blind, Placebo-Controlled, Multicenter AIO Phase II Trial (LICC). Oncoimmunology (2020) 9(1):1806680. doi: 10.1080/2162402X.2020.1806680 32923171 PMC7458621

[166] Yu S Wang C Yu J Wang J Lu Y Zhang Y. Injectable Bioresponsive Gel Depot for Enhanced Immune Checkpoint Blockade. Adv Mater (2018) 30(28):e1801527. doi: 10.1002/adma.201801527 29786888

[167] Wilson DR Sen R Sunshine JC Pardoll DM Green JJ Kim YJ. Biodegradable STING Agonist Nanoparticles for Enhanced Cancer Immunotherapy. Nanomedicine (2018) 14(2):237–46. doi: 10.1016/j.nano.2017.10.013 PMC6035751 29127039

[168] Buss CG Bhatia SN. Nanoparticle Delivery of Immunostimulatory Oligonucleotides Enhances Response to Checkpoint Inhibitor Therapeutics. Proc Natl Acad Sci U S A (2020) 117(24):13428–36. doi: 10.1073/pnas.2001569117 PMC7306768 32493746

[169] Reinhard K Rengstl B Oehm P Michel K Billmeier A Hayduk N. An RNA Vaccine Drives Expansion and Efficacy of Claudin-CAR-T Cells Against Solid Tumors. Science (2020) 367(6476):446–53. doi: 10.1126/science.aay5967 31896660

[170] Kreiter S Vormehr M van de Roemer N Diken M Löwer M Diekmann J. Mutant MHC Class II Epitopes Drive Therapeutic Immune Responses to Cancer. Nature (2015) 520(7549):692–6. doi: 10.1038/nature14426 PMC4838069 25901682

[171] Sahin U Derhovanessian E Miller M Kloke BP Simon P Löwer M. Personalized RNA Mutanome Vaccines Mobilize Poly-Specific Therapeutic Immunity Against Cancer. Nature (2017) 547(7662):222–6. doi: 10.1038/nature23003 28678784

[172] Ishihara J Fukunaga K Ishihara A Larsson HM Potin L Hosseinchi P. Matrix-Binding Checkpoint Immunotherapies Enhance Antitumor Efficacy and Reduce Adverse Events. Sci Transl Med (2017) 9(415):1. doi: 10.1126/scitranslmed.aan0401 29118259

[173] Zinger A Koren L Adir O Poley M Alyan M Yaari Z. Collagenase Nanoparticles Enhance the Penetration of Drugs Into Pancreatic Tumors. ACS Nano (2019) 13(10):11008–21. doi: 10.1021/acsnano.9b02395 PMC6837877 31503443

[174] Le QV Suh J Oh YK. Nanomaterial-Based Modulation of Tumor Microenvironments for Enhancing Chemo/Immunotherapy. AAPS J (2019) 21(4):64. doi: 10.1208/s12248-019-0333-y 31102154

[175] Bockamp E Rosigkeit S Siegl D Schuppan D. Nano-Enhanced Cancer Immunotherapy: Immunology Encounters Nanotechnology. Cells (2020) 9(9):9–:10. doi: 10.3390/cells9092102 PMC7565449 32942725

[176] Ou W Thapa RK Jiang L Soe ZC Gautam M Chang JH. Regulatory T Cell-Targeted Hybrid Nanoparticles Combined With Immuno-Checkpoint Blockage for Cancer Immunotherapy. J Control Release (2018) 281:84–96. doi: 10.1016/j.jconrel.2018.05.018 29777794

[177] Schumacher Ton N Kesmir C van Buuren Marit M. Biomarkers in Cancer Immunotherapy. Cancer Cell (2015) 27(1):12–4. doi: 10.1016/j.ccell.2014.12.004 25584891

[178] Kappel C Seidl C Medina-Montano C Schinnerer M Alberg I Leps C. Density of Conjugated Antibody Determines the Extent of Fc Receptor Dependent Capture of Nanoparticles by Liver Sinusoidal Endothelial Cells. ACS Nano (2021) 15(9):15191–209. doi: 10.1021/acsnano.1c05713 34431291

[179] Heineke MH van Egmond M. Immunoglobulin A: Magic Bullet or Trojan Horse? Eur J Clin Invest (2017) 47(2):184–92. doi: 10.1111/eci.12716 28024097

[180] Wen YM Shi Y. Immune Complex Vaccination. Curr Top Microbiol Immunol (2019) 423:95–118. doi: 10.1007/82_2019_153 30790078

[181] Sadiki A Vaidya SR Abdollahi M Bhardwaj G Dolan ME Turna H. Site-Specific Conjugation of Native Antibody. Antib Ther (2020) 3(4):271–84. doi: 10.1093/abt/tbaa027 PMC7906296 33644685

[182] Lin X O'Reilly Beringhs A Lu X. Applications of Nanoparticle-Antibody Conjugates in Immunoassays and Tumor Imaging. AAPS J (2021) 23(2):43. doi: 10.1208/s12248-021-00561-5 33718979 PMC7956929

[183] Bros M Nuhn L Simon J Moll L Mailänder V Landfester K. The Protein Corona as a Confounding Variable of Nanoparticle-Mediated Targeted Vaccine Delivery. Front Immunol (2018) 9:1760. doi: 10.3389/fimmu.2018.01760 30116246 PMC6082927

[184] Bednarczyk M Medina-Montano C Fittler FJ Stege H Roskamp M Kuske M. Complement-Opsonized Nano-Carriers Are Bound by Dendritic Cells (DC) *via* Complement Receptor (CR)3, and by B Cell Subpopulations *via* CR-1/2, and Affect the Activation of DC and B-1 Cells. Int J Mol Sci (2021) 22(6):1. doi: 10.3390/ijms22062869 PMC8001596 33799879

[185] Li M Jiang S Simon J Paßlick D Frey ML Wagner M. Brush Conformation of Polyethylene Glycol Determines the Stealth Effect of Nanocarriers in the Low Protein Adsorption Regime. Nano Lett (2021) 21(4):1591–8. doi: 10.1021/acs.nanolett.0c03756 PMC8023711 33560851

[186] Holm R Douverne M Weber B Bauer T Best A Ahlers P. Impact of Branching on the Solution Behavior and Serum Stability of Starlike Block Copolymers. Biomacromolecules (2019) 20(1):375–88. doi: 10.1021/acs.biomac.8b01545 30475598

